# Alterations in the Genomic Distribution of 5hmC in In Vivo Aged Human Skin Fibroblasts

**DOI:** 10.3390/ijms22010078

**Published:** 2020-12-23

**Authors:** Paulina Kołodziej-Wojnar, Joanna Borkowska, Zofia Wicik, Anna Domaszewska-Szostek, Jacek Połosak, Marta Cąkała-Jakimowicz, Olga Bujanowska, Monika Puzianowska-Kuznicka

**Affiliations:** 1 Department of Geriatrics and Gerontology, Medical Centre of Postgraduate Education, 01-813 Warsaw, Poland; pkolodziej@imdik.pan.pl; 2 Department of Human Epigenetics, Mossakowski Medical Research Centre, PAS, A. Pawinskiego 5, 02-106 Warsaw, Poland; jborkowska@imdik.pan.pl (J.B.); zofiawicik@gmail.com (Z.W.); adomaszewska@imdik.pan.pl (A.D.-S.); jpolosak@imdik.pan.pl (J.P.); mcjakimowicz@imdik.pan.pl (M.C.-J.); obujanowska@imdik.pan.pl (O.B.); 3 Institute of Medical Science, Faculty of Medicine, Collegium Medicum, Cardinal Stefan Wyszynski University in Warsaw, 01-938 Warsaw, Poland

**Keywords:** dermal fibroblasts, aging, epigenetic drift, 5-hydroxymethylcytosine (5hmC), regions differentially hydroxymethylated with age (DHMRs), ten-eleven translocation methylcytosine dioxygenase (TET) enzymes

## Abstract

5-Hydroxymethylcytosine (5hmC) is a functionally active epigenetic modification. We analyzed whether changes in DNA 5-hydroxymethylation are an element of age-related epigenetic drift. We tested primary fibroblast cultures originating from individuals aged 22–35 years and 74–94 years. Global quantities of methylation-related DNA modifications were estimated by the dot blot and colorimetric methods. Regions of the genome differentially hydroxymethylated with age (DHMRs) were identified by hMeDIP-seq and the MEDIPS and DiffBind algorithms. Global levels of DNA modifications were not associated with age. We identified numerous DHMRs that were enriched within introns and intergenic regions and most commonly associated with the H3K4me1 histone mark, promoter-flanking regions, and CCCTC-binding factor (CTCF) binding sites. However, only seven DHMRs were identified by both algorithms and all of their settings. Among them, hypo-hydroxymethylated DHMR in the intron of Rab Escort Protein 1 (*CHM*) coexisted with increased expression in old cells, while increased 5-hydroxymethylation in the bodies of Arginine and Serine Rich Protein 1 (*RSRP1*) and Mitochondrial Poly(A) Polymerase (*MTPAP*) did not change their expression. These age-related differences were not associated with changes in the expression of any of the ten-eleven translocation (TET) enzymes or their activity. In conclusion, the distribution of 5hmC in DNA of in vivo aged human fibroblasts underwent age-associated modifications. The identified DHMRs are, likely, marker changes.

## 1. Introduction

Skin aging does not shorten life but can significantly reduce its quality. Intrinsic aging of the skin is associated with a reduced regenerative potential of keratinocytes, thinning of the epidermis, and atrophy of the dermis resulting from fibroblast senescence and overexpression of enzymes degrading the collagen matrix [1,2]. An increasing amount of evidence shows that changes in the function of subcutaneous adipose tissue may be one of the regulators of skin aging [3].

The epigenome co-regulates activities of the genome without changing its sequence and undergoes aging-related drift. The role of this phenomenon in aging is crucial, as epigenetic modifications are subject to environmentally induced changes, and the environment seems to be the most important factor affecting the length of life [4,5]. Although most aging-related changes affecting the epigenome are random, some of them are universal, and sets of such changes have been used to create epigenetic clocks that predict chronological age with high accuracy [6,7,8]. One of the epigenetic modifications, 5-hydroxymethylcytosine (5hmC), appears during the processes of passive demethylation of 5-methylcytosine (5mC) or active demethylation by ten-eleven translocation methylcytosine dioxygenases (TETs) [9,10]. 5hmC is not only an intermediate product of demethylation, but mounting evidence points to its role in the regulation of gene expression. For example, the hydroxymethylation of promoters and gene bodies is associated with increased expression [11,12,13]. A proposed mechanism of such an action of 5hmC is preventing transcriptional repressors from binding [14]. In addition, 5hmC supports gene activation by engaging Methyl-CpG Binding Protein 2 (MeCP2), which modifies chromatin structure [15,16]. Therefore, 5hmC and TETs may affect chromatin accessibility for transcription factors [17]. In contrast to many studies regarding the association and involvement between DNA methylation and aging, only limited data have been published with respect to the association between DNA 5-hydroxymethylation and aging. For example, a longitudinal study of DNA isolated from mouse blood cells showed that the global level of 5hmC decreases with age in these cells [18]. Similarly, the global level of 5hmC in human blood genomic DNA systematically decreases as the blood donor’s age increases [19]. In contrast, a significant rise in 5hmC is observed in the aging mouse cerebellum and liver [20,21].

Therefore, aging-associated changes in global 5hmC content seem to be cell- and organ-specific, and it cannot be excluded that some are species-specific. Under such circumstances, an appropriate model for studying molecular mechanisms of human skin aging is human skin-derived cell culture. The majority of aging and senescence studies have been conducted on established cell lines or in vitro-aged cells. However, such cells do not present the same features as cells aged in vivo [22,23,24]. As we wished to obtain information closely reflecting the in vivo state, we used dermis samples from sun-protected skin areas of people of different ages and primary cultures of fibroblasts isolated from these samples. We analyzed the global content of cytosine modifications and identified several differentially hydroxymethylated regions (DHMRs) associated with dermal fibroblast aging.

## 2. Results

### 2.1. Cytosine Modifications in the Dermis and Fibroblasts

**Figure 1:**
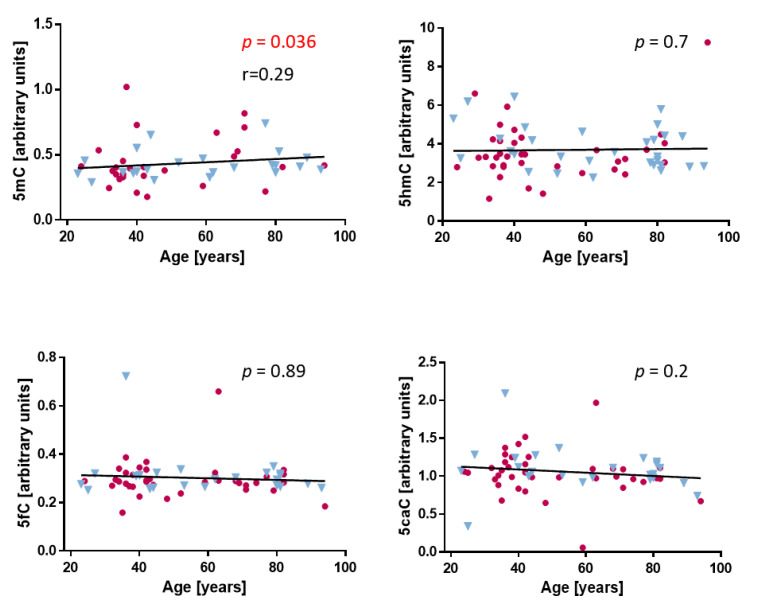
Levels of 5-methylcytosine (5mC), 5-hydroxymethylcytosine (5hmC), 5-formylcytosine (5fC) and 5-carboxylcytosine (5caC) in human dermis. Levels of DNA methylation and demethylation derivatives in the dermis evaluated with the dot-blot method. The statistical analysis was performed with the Mann–Whitney *U*-test and Spearman’s correlation test. Purple circles: women. Blue triangles: men.

We first checked levels of 5mC, 5hmC, and further derivatives of demethylation 5-formylcytosine (5fC) and 5-carboxylcytosine (5caC) in the whole dermis (Supplementary Figure S1A). We found that they were similar in women and men, so further calculations were performed for both sexes together. We observed a weak correlation between 5mC and age (r = 0.29, *p* = 0.036). Levels of 5hmC, 5fC, and 5caC did not correlate with age (Figure 1). To confirm this result, we counted the mean fluorescence intensity of 100 nuclei stained for the presence of 5mC and 5hmC in the dermis samples using a confocal microscope and the ZEN 2012 version BLUE program (Supplementary Figure S2). This analysis also did not detect any age-related differences in global 5hmC amounts or the 5hmC/5mC ratio.

**Figure 2:**
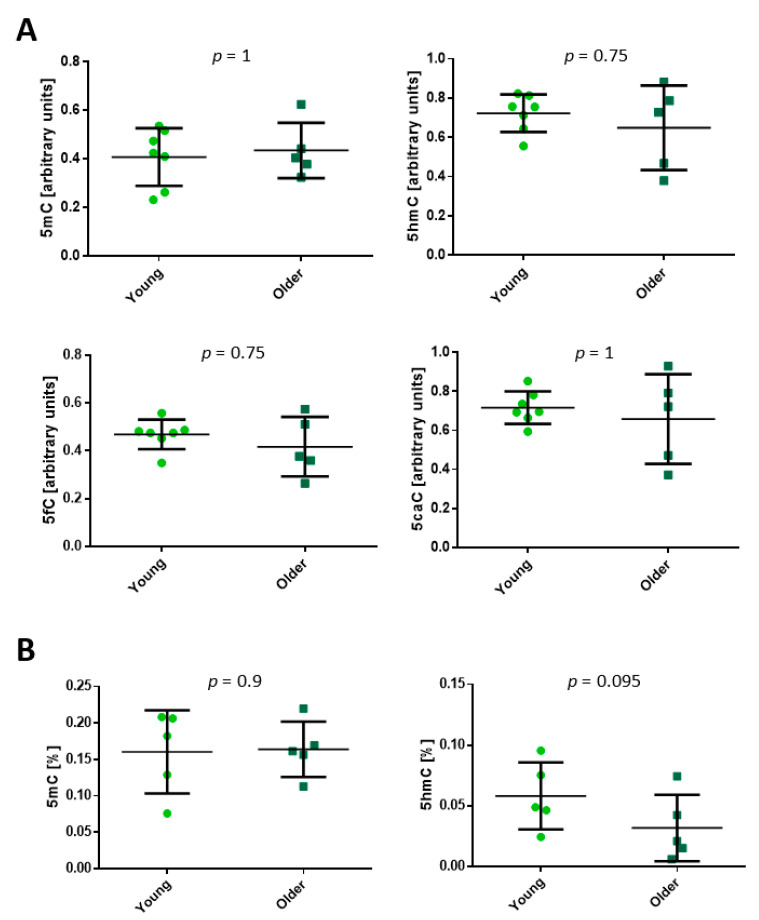
Levels of 5-methylcytosine (5mC), 5-hydroxymethylcytosine (5hmC), 5-formylcytosine (5fC) and 5-carboxylcytosine (5caC) in human dermal fibroblasts. (**A**) Levels of DNA methylation and methylation derivatives in dermal fibroblasts evaluated with the dot-blot method. (**B**) Levels of DNA methylation and hydroxymethylation in dermal fibroblasts evaluated with the colorimetric method. The statistical analysis was performed with the Mann–Whitney *U*-test. Young: 22–35 years old. Older: 74–94 years old.

In fibroblasts isolated from young and older individuals’ dermis, we did not detect differences in total 5mC, 5hmC, 5fC, or 5caC content as evaluated by the dot-blot method (Supplementary Figure S1B and Figure 2A). This result was confirmed by the colorimetric evaluation of global 5mC and 5hmC, as it also did not reveal age-related differences (Figure 2B).

**Figure 3:**
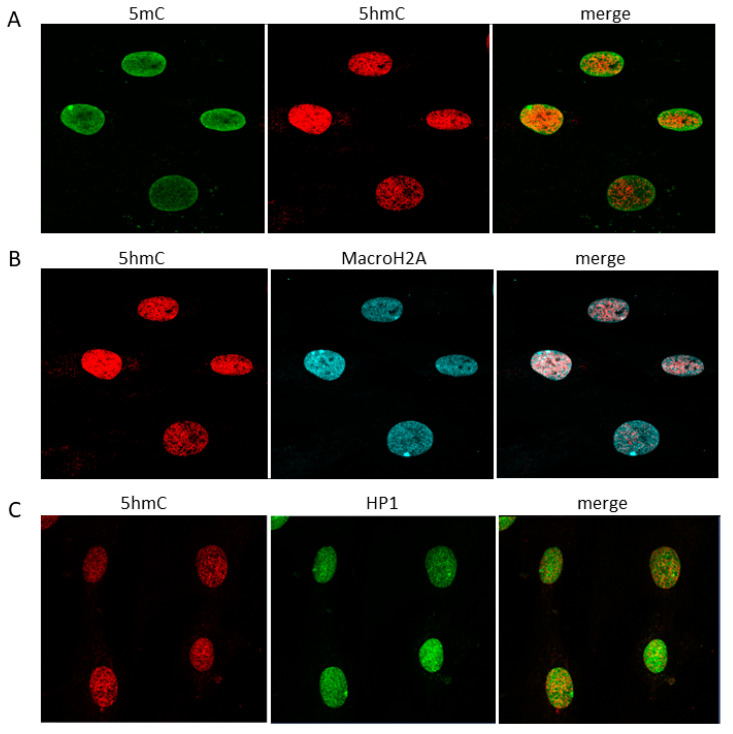
Immunofluorescence staining for the presence of 5-methylcytosine (5mC), 5-hydroxymethylcytosine (5hmC), and heterochromatin markers in human fibroblasts. (**A**) Immunostaining for the presence of 5mC and 5hmC. (**B**) Co-localization of 5hmC and MacroH2A histone variant. (**C**) Co-localization of 5hmC and heterochromatin protein 1 (HP1).

We also checked the nuclear localization of 5mC and 5hmC and found that they only partially overlapped (Figure 3A). Immunostaining of 5hmC with the heterochromatin markers MacroH2A histone variant or heterochromatin protein 1 (HP1) showed that, to a large extent, 5hmC localization differed from that of the heterochromatin markers (Figure 3B,C).

### 2.2. Age-Related Differential 5-Hydroxymethylation in Fibroblasts

The lack of age-related differences in the quantity of global 5hmC does not exclude age-related changes in 5hmC distribution. Therefore, using two different algorithms but with the same set of DNA window sizes (wide, approximately 450 bp and narrow, approximately 50 bp) and statistical methods, we searched for age-related differentially hydroxymethylated regions (DHMRs) in DNA isolated from fibroblasts of five young and five older individuals. 

**Figure 4:**
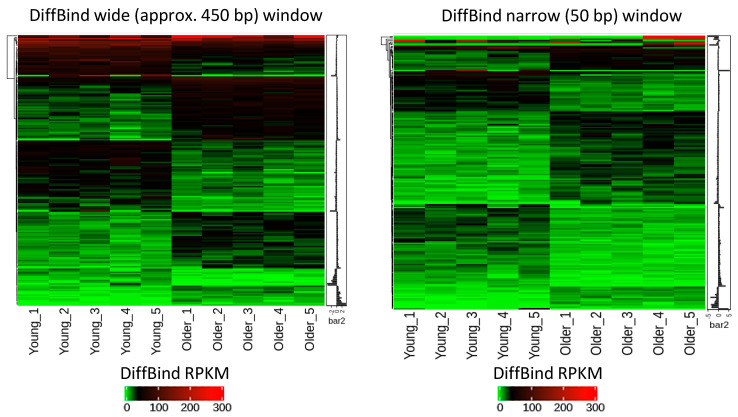

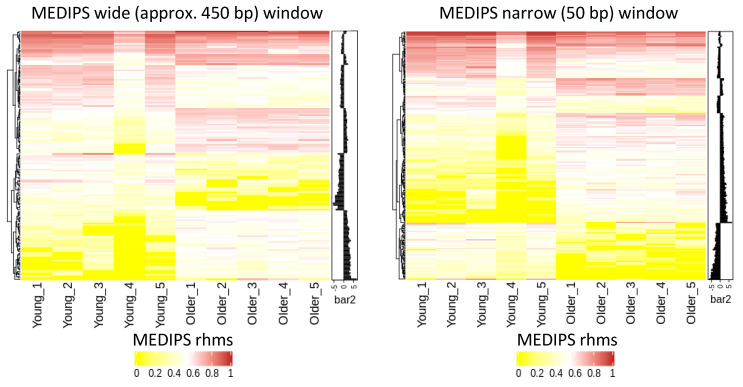
Heatmaps of age-associated differentially hydroxymethylated regions (DHMRs) in the DNA of human fibroblasts obtained from young (*n* = 5, 22–35 years) and older (*n* = 5, 74–94 years) individuals. The 5-hydroxymethylcytosine (5hmC) distribution was analyzed using hMeDIP-seq. RPKM: reads per kilobase million, rhms: relative hydroxymethylation score.

A DiffBind analysis performed for the wide window identified 190 DHMRs, including 102 that were hyper- and 88 that were hypo-hydroxymethylated, while the analysis of the narrow window identified 186 DHMRs, including 97 hyper- and 89 hypo-hydroxymethylated (Figure 4, Supplementary Table S1). Eighty-five regions were common to both windows. The MEDIPS analysis of the wide window identified 161 DHMRs, including 83 hyper- and 78 hypo-hydroxymethylated, and the analysis of the narrow window identified 228 DHMRs, including 121 hyper- and 107 hypo-hydroxymethylated (Figure 4, Supplementary Table S1). Sixty-seven regions were common to both windows.

**Figure 5:**
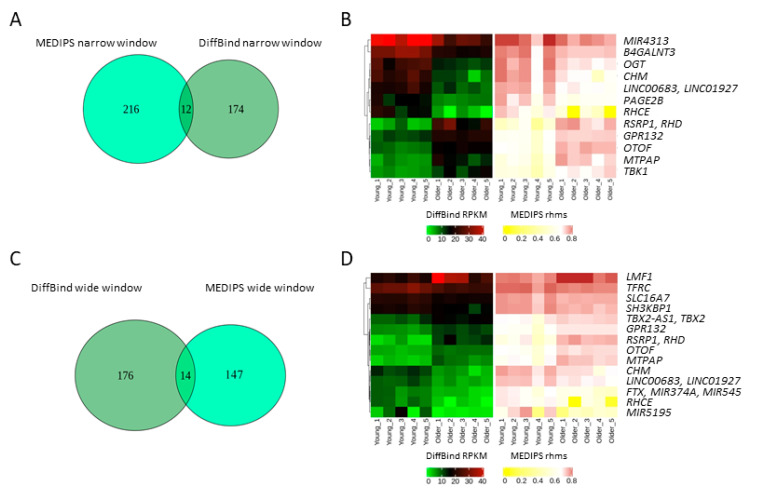
Age-associated differentially hydroxymethylated regions (DHMRs) identified by DiffBind and MEDIPS with the same window size. (**A**) Venn diagram and (**B**) Heatmaps of DHMRs identified by a narrow window analysis. (**C**) Venn diagram and (**D**) Heatmaps of DHMRs identified by a wide window analysis. RPKM: reads per kilobase million, rhms: relative hydroxymethylation score.

Next, we compared the DHMRs identified by both algorithms with the same window size. Only 14 DHMRs were common for the wide window, and only 12 DHMRs were common for the narrow window despite very similar analysis settings (Figure 5).

**Figure 6:**
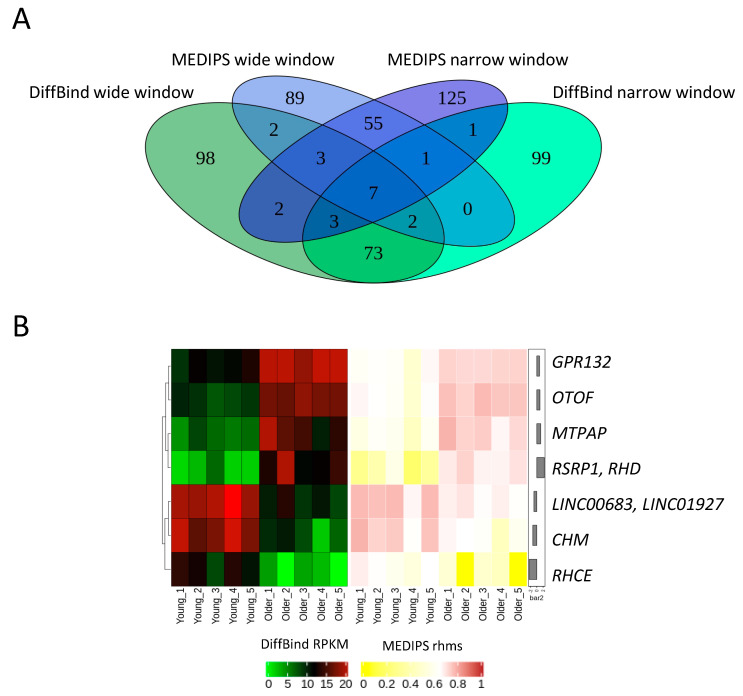
Age-associated differentially hydroxymethylated regions (DHMRs) in human fibroblasts common for all analyses. (**A**) Venn diagram of overlapping DHMRs. (**B**) Heatmap of overlapping DHMRs and their associated genes. RPKM: reads per kilobase million, rhms: relative hydroxymethylation score.

Only seven DHMRs were identified by both MEDIPS and DiffBind analyses and for both window sizes. These regions were associated with *CHM* (Rab Escort Protein 1), *RHCE* (Rh Blood Group CcEe Antigens), *LINC00683* (Long Intergenic Non-Protein Coding RNA 683), *LINC01927* (Long Intergenic Non-Protein Coding RNA 1927), *GPR132* (G Protein-Coupled Receptor 132), *RSRP1* (Arginine and Serine Rich Protein 1), *RHD* (Rh Blood Group D Antigen), *OTOF* (Otoferlin) and *MTPAP* (Mitochondrial Poly(A) Polymerase) genes. All were situated within genes, mostly in introns (Figure 6).

**Figure 7:**
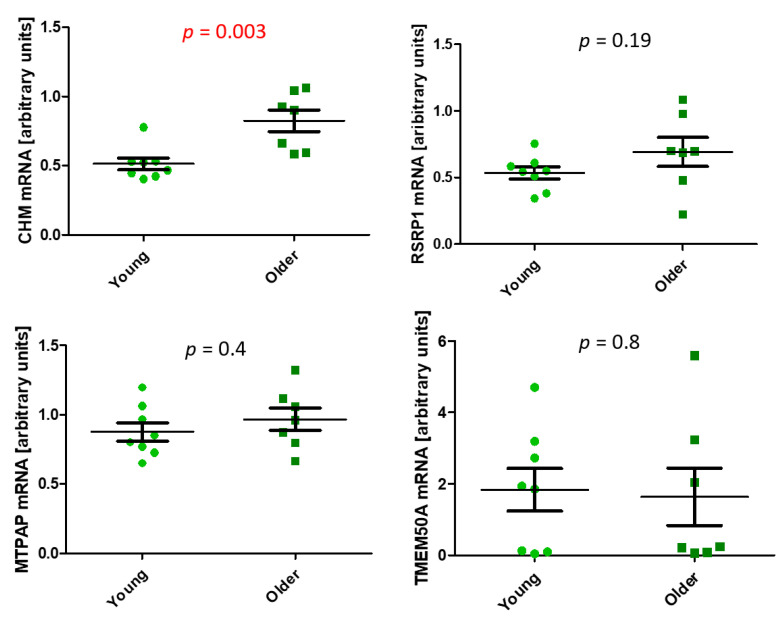
RT-PCR evaluation of mRNA expression of genes associated with age-related differentially hydroxymethylated regions (DHMRs) identified by both DiffBind and MEDIPS algorithms and both wide (approximately 450 bp) and narrow (50 bp) window sizes. The statistical analysis was performed with the Student’s *t*-test. Young (22–35 years), older (74–94 years).

To verify whether the presence of hyper- or hypo-hydroxymethylated DHMRs corresponded to differences in expression of their associated genes, we performed a real-time PCR analysis. Blood type-related genes were not analyzed, as they are not expressed in dermal fibroblasts. Instead, we analyzed the expression of *TMEM50A* (Transmembrane Protein 50A), which was identified by the GREAT tool [25] as a gene associated with the highest number of DHMRs. *CHM* expression was higher in older than younger individuals (*p* = 0.003) (Figure 7). No age-associated differences were observed in the expression of *RSRP1*, *MTPAP*, or *TMEM50A*. We did not detect *OTOF* mRNA. 

We annotated the identified DHMRs with regard to the presence of CpG islands, genic features, histone marks, and regulatory features. In all analyses, most DHMRs were situated in introns or intergenic regions. H3K4me1 was the most common histone mark associated with the DHMRs. Promoter-flanking regions and CCCTC-binding factor (CTCF) binding sites were the most common regulatory features. 

**Figure 8:**
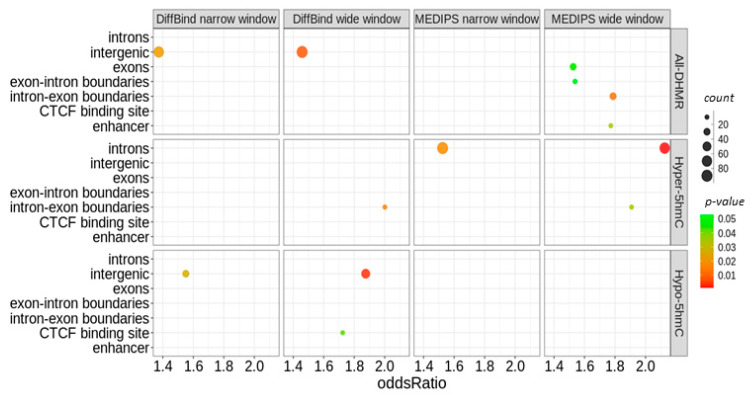
Enrichment analysis of age-associated differentially hydroxymethylated regions (DHMRs). The statistical analysis was performed with Fisher’s exact test and the LOLA package. Results are presented for *p*-values without the false discovery rate (FDR) correction.

The statistical analysis of the DiffBind-identified DHMRs showed that they were significantly enriched in intergenic regions and were mostly hypo-hydroxymethylated compared with random regions generated from all regions hydroxymethylated in fibroblasts. In addition, CTCF-binding sites were enriched among hypo-hydroxymethylated DHMRs identified in the wide window analysis. In turn, all MEDIPS-identified DHMRs were enriched in exon, intron-exon, and exon-intron boundaries and enhancers. A separate analysis of hypo- and hyper-hydroxymethylated MEDIPS-identified DHMRs showed that hyper-hydroxymethylated DHMRs were significantly enriched in introns and intron-exon boundaries (Figure 8). However, none of these enrichments passed the FDR correction.

**Figure 9:**
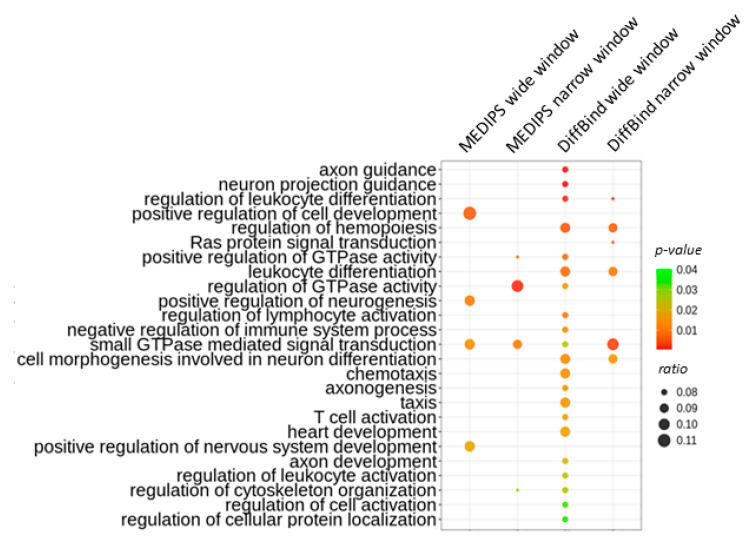
Biological processes most significantly associated with genes linked with age-associated differentially hydroxymethylated regions (DHMRs). The overrepresentation test of the Gene Ontology pathways was performed with the ClusterProfiler Package. Results are presented for *p*-values without the FDR correction.

Finally, we annotated the identified DHMRs to genes and looked for pathways potentially affected by aging-related changes in the 5hmC level. An analysis with the false discovery rate (FDR) correction did not reveal any enriched pathways. However, to reduce the risk of a false-negative result, we also performed an analysis without this correction. We then found enrichment in pathways involved in GTPase activity and signaling as well as other fibroblast-related pathways. However, we also found enrichment in pathways not related to fibroblast functions (Figure 9).

### 2.3. Age-Related Differences in TET Expression in the Dermis and Fibroblasts

5hmC is a product of either passive demethylation of 5mC or active demethylation by TET1, TET2, and TET3. Therefore, we evaluated these enzymes’ expression and showed that TET1 and TET3 mRNA levels were higher in the entire dermis of women than in men (*p* < 0.0001 and *p* = 0.03), but TET2 mRNA was similar between the sexes. No age-related differences were observed in the mRNA expression of any of the TET enzymes either in the entire tested group or in men or women separately (Supplementary Figure S3A–C). The TET2 protein level was not associated with age. We did not evaluate the TET1 or TET3 proteins’ expression because specific signals in the dermis were too low, despite using three different anti-TET1 and two anti-TET3 antibodies. In contrast, the levels of these proteins were high in the epidermis (Supplementary Figure S3D–F). 

No age-related differences were observed in the expression of TETs either at the mRNA or protein levels in primary fibroblasts (Supplementary Figure S4). In addition, no significant differences in the activity of TET enzymes were detected (Supplementary Figure S5).

We did not observe correlations between 5mC, 5hmC, 5fC, or 5caC and the expression of any of the TET enzymes in either the entire dermis or fibroblasts.

## 3. Discussion

This study showed that global levels of DNA demethylation products do not change in the intrinsically aging sun-protected human dermis or in in vivo aging human fibroblasts. However, the global level of 5hmC was estimated with the dot-blot and colorimetric methods, both widely used but not adapted to detect subtle changes, which could, therefore, be overlooked. Nevertheless, the distribution of 5hmC in DNA of fibroblasts changed with age. We identified several differentially hydroxymethylated sites; approximately half of them were hyper, and half were hypo-hydroxymethylated. This finding agrees with data regarding single differentially hydroxymethylated CpG sites in human bone marrow mesenchymal stem cells, which are almost as often hyper- as hypo-hydroxymethylated in cells of older donors [26]. However, contrasting data were obtained by other authors with regard to global 5hmC changes. The aging of human peripheral blood mononuclear cells is associated with a significant decrease in global 5hmC [27]. Accumulation of 5caC and hypo-hydroxymethylation in DNA was also detected in a longitudinal study in mice [18]. In turn, evidence indicates that aging is associated with an increase in global 5hmC levels in the human cerebrum and cerebellum, as well as in the mouse hippocampus [28,29]. In contrast, others have not reported aging-related changes in the mouse brain [30]. Based on these and other data, we hypothesized that aging-related changes in global DNA 5-hydroxymethylation are cell- and tissue-specific. 

Changes in hydroxymethylation result not only from passive but also from active DNA demethylation, and aging-associated changes in the global amount of 5hmC can be related to changes in expression of the TET enzymes catalyzing active demethylation. We did not detect such changes in the human dermis or dermal fibroblasts. In contrast, other authors have reported that TET2 expression is lower in full-thickness skin of older mice than in the skin of younger animals, while the expression of TET1 is similar [31]. Older age is associated with lower TET1 and TET3 mRNA levels in human blood cells [27]. Moreover, some researchers have shown that TET1 and TET2 mRNA levels are lower in the hippocampus of aged mice than their young counterparts, whereas others have claimed that there are no age-related TET1, TET2, or TET3 mRNA changes in this brain structure [28,30,32]. Based on limited data, we hypothesized that age-related changes in TET expression are also cell- and tissue-specific, especially that these enzymes are not uniformly expressed (https://www.proteinatlas.org). It is also plausible that the inconsistent results regarding global content of DNA demethylation derivatives and expression of TETs are a result of imperfect methodology or different study designs. Another issue is that no correlation was detected between the global content of 5hmC and the expression of either TET [27,28] in our study and other studies. At least two explanations can be presented for this observation. First, not only the level of expression but also the activity of each of the TET enzymes, which depends on many factors, as well as the availability of the necessary co-factors, can play a role in this phenomenon [33,34,35,36]. Second, all three TETs produce 5hmC, but none of them prevail to the extent of allowing detection with available methodology.

Despite the failure to detect changes in the global amount of 5hmC, we detected changes in the distribution of this DNA modification. Because our additional goal was to verify if different methods of analyzing the same raw data yield the same or divergent results, we searched for differentially hydroxymethylated regions using two different algorithms. We showed that only about 10% of the DHMRs were identified by both DiffBind and MEDIPS when the same or similar settings and the same statistical methods were used. This result points to the critical role of selecting the proper algorithm and settings for the hydroxymethylation analysis. Moreover, the lack of a standardized method may make it difficult or even impossible to compare results between laboratories.

We showed that DHMRs were mostly located in intergenic regions and introns of dermal fibroblasts and were most commonly associated with promoter-flanking regions, CTCF-binding sites, and the H3K4me1 histone mark. A DiffBind analysis showed that hypo-hydroxymethylated DHMRs were significantly enriched in intergenic regions and CTCF-binding sites compared to the randomly generated regions. In turn, MEDIPS identified significantly enriched hyper-hydroxymethylated DHMRs in introns and intron–exon boundaries. None of these enrichments have passed the FDR correction. An analysis of individual CpG sites in bone marrow mesenchymal stem cells obtained from eleven young and six older patients showed that 1631 sites were differentially hydroxymethylated, and such sites were preferentially located in intergenic regions, but, when located in the gene, they were preferably present in gene bodies than in promoter regions. Moreover, differentially hydroxymethylated cytosine residues were associated with the active histone mark H3k4me1 [26], which was supported by our findings. These authors also found a significant association with enhancers. 

Some of the identified DHMR-associated genes are expressed ubiquitously, while the expression of others is restricted to particular tissues or organs, including or excluding fibroblasts. Moreover, they are involved not only in pathways related to fibroblast function but also in other pathways. Therefore, we hypothesized that the identified DHMRs should be treated as aging markers rather than functional indicators of the direction of aging-related changes in skin fibroblasts. To further support our hypothesis, we analyzed the expression of DHMR-associated genes identified by both algorithms and all settings that should be active in fibroblasts. We found that the 5hmC level was lower in the intron of the *CHM* gene, while this gene expression was higher in older than in younger individuals. As previously published data indicate that 5hmC level is increased in gene bodies of actively transcribed genes, our results regarding *CHM* were not in agreement with these data [11]. In addition, no age-associated difference in expression of *RSRP1* and *MTPAP* was observed, while 5-hydroxymethylation in their bodies increased in old cells, a constellation also not in agreement with previous data [11]. Finally, the expression of *TMEM50A* associated with the highest number of DHMRs was also similar in young and old cells. However, in this case, DHMRs identified in silico as potentially interacting with this gene were located quite distantly and may not have had an actual effect on its activity. Alternatively, the effects of many DHMRs could abolish each other. Therefore, further experiments are needed to determine the kind of functional consequences that occur due to hydroxymethylation changes.

Our study had some limitations. First, global DNA modification levels were assessed by the dot blot and colorimetric methods, which are not precise, and slight differences could go unnoticed. Second, we compared cultures from only five young and five older donors in our search for DHMRs. However, it should be emphasized that our model had two advantages. First, we studied human, not animal cells. Second, the cells originated from young and age-advanced individuals and were cultured for a limited time; therefore, they more closely resembled the aging of a living human cells than cultures aged *in vitro*, which are commonly used in aging studies.

In conclusion, we showed for the first time that aging of human dermis and dermal fibroblasts is not associated with marked changes in global amounts of 5mC-derived intermediates of demethylation or expression of TET enzymes. However, aging was associated with a significant change in the distribution of 5-hydroxymethylcytosine in genomic DNA. Whether or not the identified DHMRs have functional consequences or are marker changes requires further investigation.

## 4. Materials and Methods

### 4.1. Dermis Samples

Skin samples were obtained from the sun-protected suprapubic or groin areas of 67 individuals (36 women and 25 men, 22–94 years) undergoing elective surgery due to reasons unrelated to this project, such as hernia or plastic surgery. All skin donors were free of local and systemic diseases that could affect the skin condition. 

### 4.2. Fibroblast Isolation and Cell Culture

Dermis samples from eight young (Y, 22–35 years) and seven older (O, 74–94 years) individuals were processed according to a previously published protocol [37], with modifications—samples were digested with dispase II (Sigma-Aldrich, St. Louis, MO, USA) at 4 °C, overnight, and then digested with collagenase IV (Sigma-Aldrich) at 37 °C, overnight. Cells from passages 4 and 5 were used in all experiments [38].

### 4.3. TET Activity Assay

Nuclear extracts were isolated from dermal fibroblasts using the EpiQuik Nuclear Extraction Kit II (EpiGentek, Farmingdale, NY, USA). The TET activity assay was performed with the colorimetric Epigenase 5mC-Hydroxylase TET Activity/Inhibition Assay Kit (EpiGentek) according to the manufacturer’s instructions. Absorbance at 450 nm and 655 nm (reference) was read in the Epoch microplate spectrophotometer (BioTek, Winooski, VT, USA).

### 4.4. Isolation of DNA and Dot-Blot Evaluation of Global DNA Modifications Content

Approximately 100 mg of the dermis was homogenized in the TissueLyser II (Qiagen, Hilden, Germany) in PBS at 4 °C, then digested with proteinase K (EurX, Gdańsk, Poland) for 16 h at 55 °C. DNA was isolated using the Tissue DNA Purification Kit (EurX). DNA was also isolated from primary fibroblast cultures using the Sherlock AX isolation kit (A&A Biotechnology, Gdynia, Poland).

DNA (100 ng) was denatured in 0.4 M NaOH and 10 mM EDTA for 10 min at 100 °C, neutralized with 2 M ammonium acetate, applied to a nitrocellulose membrane on the Bio-Dot instrument (Bio-Rad, Hercules, CA, USA), and UV-crosslinked. Dot-blots were blocked and then incubated with anti-5mC (1:2000, Abcam, Cambridge, UK) anti-5hmC, anti-5fC, or anti-5CaC antibodies (1:10,000, Active-Motif, La Hulpe, Belgium). Incubation with an anti-ssDNA antibody served as a loading control (1:1000, Enzo, New York, NY, USA). Next, the blots were washed in TBST and incubated with an appropriate secondary antibody. Signals were detected using the enhanced chemiluminescent (Bio-Rad) and GeneGnome Chemiluminescence imaging systems (Syngene, Cambridge, UK). The quantitative analysis was performed using Image Studio™ Lite software (LI-COR, Lincoln, NE, USA).

### 4.5. Colorimetric Evaluation of Global 5mC and 5hmC Content 

Global 5mC level was measured with the colorimetric Methylated DNA Quantification Kit (Abcam), and 5hmC level was measured with colorimetric Global DNA Hydroxymethylation Assay Kit (Abcam) according to the manufacturer’s protocols. DNA (100 ng) from each fibroblast culture was used in each test. Absorbance at 450 nm was evaluated using the Epoch Microplate Spectrophotometer (Bio Tek).

### 4.6. Isolation of Proteins and Immunoblot

For total protein isolation, a 100 mg dermis sample was homogenized in 500 µL RIPA buffer supplemented with a protease inhibitor mix (Roche, Mannheim, Germany) in the TissueLyser II (Qiagen) at 4 °C for 5 min, and centrifuged at 14,000× *g* for 20 min at 4 °C. Approximately one million fibroblast cells suspended in 500 µL RIPA buffer supplemented with the protease inhibitor mix were incubated on ice for 10 min with vortexing every 2 min and then centrifuged at 400× *g* for 20 min at 4 °C. The protein concentration was determined using the BCA Protein Assay kit (Thermo Scientific, Waltham, MA, USA). Soluble proteins (30 µg of dermis extract or 20 µg of fibroblasts extract) were used for electrophoresis and transferred to membranes according to standard procedures. The membranes were blocked and incubated with anti-TET1 (1:1000, Thermo Fisher, Rockford, IL, USA), anti-TET2 (1:1000, Active Motif), anti-TET3 (1:1000, Abiocode, Agoura Hills, CA, USA) or anti-GAPDH (1:1000, Santa Cruz Biotechnology, Dallas, TX, USA) primary antibodies, followed by washing in TBST and incubation with appropriate secondary antibodies. Expression of GAPDH served as internal control. Signals were detected as described above. 

### 4.7. Immunofluorescence

Skin fragments were deep-frozen and cut using the Cryocut 1800 (Leica, Reichert-Jung, Germany) into 20 µm slices that were placed onto glass slides covered with poly-α-lysine. Isolated skin fibroblasts (0.1 × 10^5^ per well) were seeded on coverslips placed in a 24-well dish. Tissue slices and fibroblasts attached to the coverslip were stained following standard procedures. In short, tissue sections and fibroblasts were fixed in 4% paraformaldehyde, washed, permeabilized with 0.5% Triton X-100, incubated in 4 N HCl, neutralized, blocked with 10% goat serum, 3% BSA, and 0.1% Triton X-100, then incubated overnight at 4 °C with anti-5mC (1:100, Abcam) and anti-5hmC (1:300, Active Motif). The fibroblasts were also stained with antibodies against the heterochromatin markers HP1 (1:100, Santa Cruz Biotechnology) and MacroH2A (1:100, Santa Cruz Biotechnology). Then, appropriate secondary antibodies (5mC: 1:2000 anti-mouse Alexa Fluor 488 (Life Technologies, Rockford, IL, USA), 5hmC: 1:2000 anti-rabbit Alexa Fluor 546 (Life Technologies) or 1:500 anti-rabbit Alexa Fluor 633 (Invitrogen, Eugene, OR, USA), HP1: 1:1000 anti-mouse Alexa Fluor 488 (Invitrogen), MacroH2A: 1:1000 anti-mouse Alexa Fluor 546 (Invitrogen)) were used. Fluorescence was analyzed using the LSM 780/ELYRA PS.1 confocal microscope (Zeiss, Jena, Germany). 

A total of 100 nuclei labeled with anti-5mC and anti-5hmC antibodies were analyzed for fluorescence intensity in dermis fragments of eight young (Y, 23–35 years) and seven older (O, 75–94 years) individuals. Mean fluorescence was calculated using the ZEN 2012 version BLUE program (Zeiss). Cells in hair follicles, sweat glands, sebaceous glands, blood vessels, as well as inflammatory infiltrates, were not evaluated.

### 4.8. RNA Isolation, cDNA Synthesis, and Real-Time PCR

RNA was isolated from 100 mg of the dermis or 10^6^ fibroblasts per isolation using the Direct-zol RNA MiniPrep kit (Zymo Research, Irvine, CA, USA), according to the manufacturer’s protocol. Reverse transcription was performed with 200 ng of total RNA using the Maxima H Minus First Strand cDNA Synthesis Kit (Thermo Fisher). Real-time PCR was performed with 1 ng template cDNA, the primers listed in Supplementary Table S2, and the LightCycler 480 Sybr Green I Master Kit (Roche) in the Light Cycler 480 Instrument II (Roche). The PCR cycles were: initial denaturation at 95 °C for 5 min, then 45 cycles of 12 s at 95 °C, 12 s at the suitable temperature (Supplementary Table S2), 12 s at 72 °C, and one melting curve cycle. All reactions were performed in duplicate. Expression of *TBP* served as an internal control. 

### 4.9. hMeDIP and Bioinformatics Analysis

DHMRs were searched for in DNA isolated from fibroblasts of five young (Y, 22–35 years) and five older (O, 74–94 years) individuals. The hMeDIP-seq service was performed by the NXT-Dx Co. (Gent, Belgium). The paired-end 50 bp sequence reads were mapped using STAR (v2.5) software. Peaks were generated using the MACS14 (Model-based Analysis of ChIP-Seq) peak caller [39]. The differential analysis of the hMeDIP-seq data was performed with the R packages DiffBind [40] and MEDIPS [41]. For DiffBind, a consensus peakset for the differential analysis was created from the 5hmC peaks identified by MACS14, and only peaks present in at least three individuals were used in further analysis. The analysis was performed in two variants—for the full length, “wide window” (mean size 450 bp) fragments and short, “narrow window” (50 bp) fragments re-centered around the strongest enrichment. The first variant corresponded to the mean length of the sequenced DNA fragments, while the second variant allowed for a more precise analysis of shorter genomic features, such as regulatory elements and intron-exon boundaries. For the MEDIPS analysis, reads were extended to 450 bp, and the genome was divided into wide (450 bp) and narrow (50 bp) window sizes. Differential analysis was performed for regions present in at least five counts in the same individual. For both packages, library size was normalized to the full library size using the TMM (trimmed mean of M-values) method. The statistical analysis was performed with the EdgeR algorithm. *p*-values were corrected using the false discovery rate (FDR) method. The threshold for the adjusted *p*-value was set to 0.1 [18].

Annotation regarding histone marks was obtained from http://www.roadmapepigenomics.org/, and annotations of regulatory features were obtained from the Ensembl Regulatory Build [42]. Genic features were obtained from the UCSC knownGene database for the hg38 human genome (https://bioconductor.org/packages/release/data/annotation/html/TxDb.Hsapiens.UCSC.hg38.knownGene.html), and Ensembl release 96 [43]. CpG annotations were obtained from the AnnotationHub Bioconductor package (https://www.bioconductor.org/packages/devel/bioc/manuals/AnnotationHub/man/AnnotationHub.pdf). The identified DHMRs were annotated with the Annotatr R package from Bioconductor. Random regions were generated by the locus overlap analysis (LOLA) R package, which provides an automatable enrichment analysis for genomic region sets [44].

Gene annotations for gene ontology were obtained from the KnownGene database and the EnsDb.Hsapiens v.86 Ensembl-based annotation package. Gene annotation of potentially regulated genes was performed with the ChiPpeakAnno [45] package and the rGREAT package [25]. The over-representation test of the Gene Ontology pathways was performed with the ClusterProfiler package. A heatmap of the DHMRs identified by the DiffBind package was generated using the Bioconductor ComplexHeatmap package for R software [46]. Graphs were prepared using ggplot2 R library [47]. 

### 4.10. Statistical Analysis

The data distributions were determined with the Shapiro–Wilk normality test. Student’s *t*-test was used to compare between groups of normally distributed data, and the Mann–Whitney U test was used for skewed distributions. Because of the skewed distribution, the correlation analysis was performed using Spearman’s rank correlation coefficient analysis. Calculations were performed in Prism 5 (GraphPad Software, Inc., San Diego, CA, USA). Linear regression analysis was conducted in the R-commander package. The differential binding affinity analysis was performed with the DiffBind R package and the EdgeR algorithm, and *p*-values and FDR values were assigned to each candidate binding site. We used EdgeR with an FDR correction for the MEDIPS package analysis. The significance of enrichment was verified with Fisher’s exact test using the LOLA R package. All 5hmC peaks identified in fibroblasts were used as a background set. The level of significance for all analyses was established at a corrected *p* < 0.05.

## Data Availability

Data available on request.

## Supplementary Materials

The following are available online at https://www.mdpi.com/1422-0067/22/1/78/s1.
