# Supplementary material for: Alterations in the Genomic Distribution of 5hmC in In Vivo Aged Human Skin Fibroblasts
Source: Int J Mol Sci. 2020 Dec 23;22(1):78. doi: 10.3390/ijms22010078 (PMC7794952; doi:10.3390/ijms22010078)
Supplement: Supplementary file 1 [file ijms-22-00078-s001.pdf]

Supplementary Materials

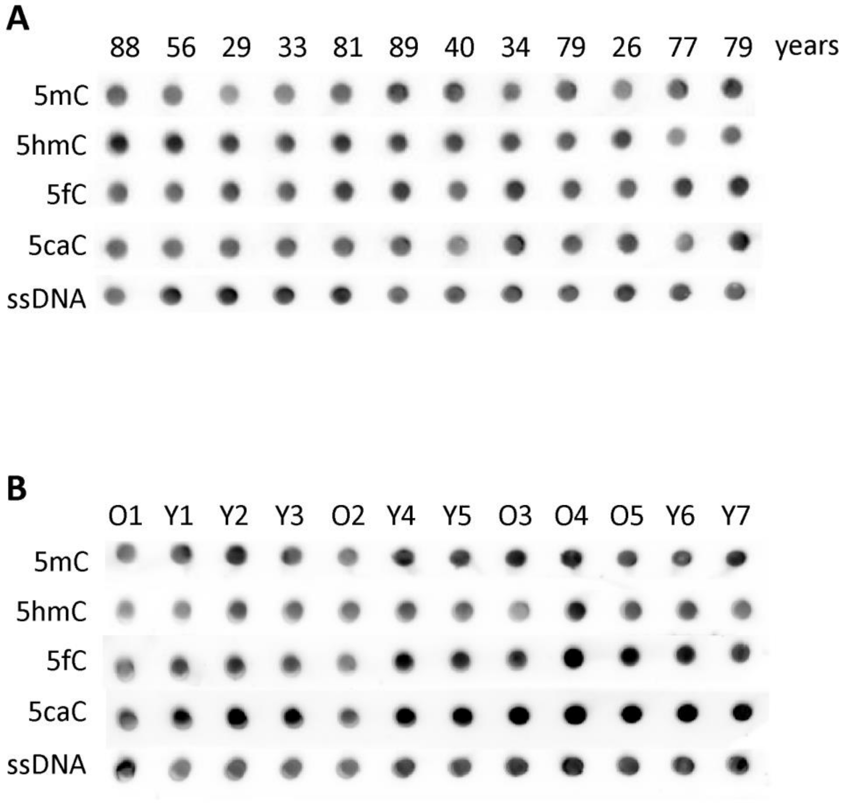

Supplementary Figure S1. Examples of dot blots evaluating global amounts of 5mC, 5hmC, 5fC, 5caC, and control ssDNA in individuals of various ages. A. Dot blots of DNA isolated from the whole human dermis. B. Dot blots of DNA isolated from primary fibroblast cultures originating from young (Y, 22–35 years) and older (O, 74–94 years) individuals.

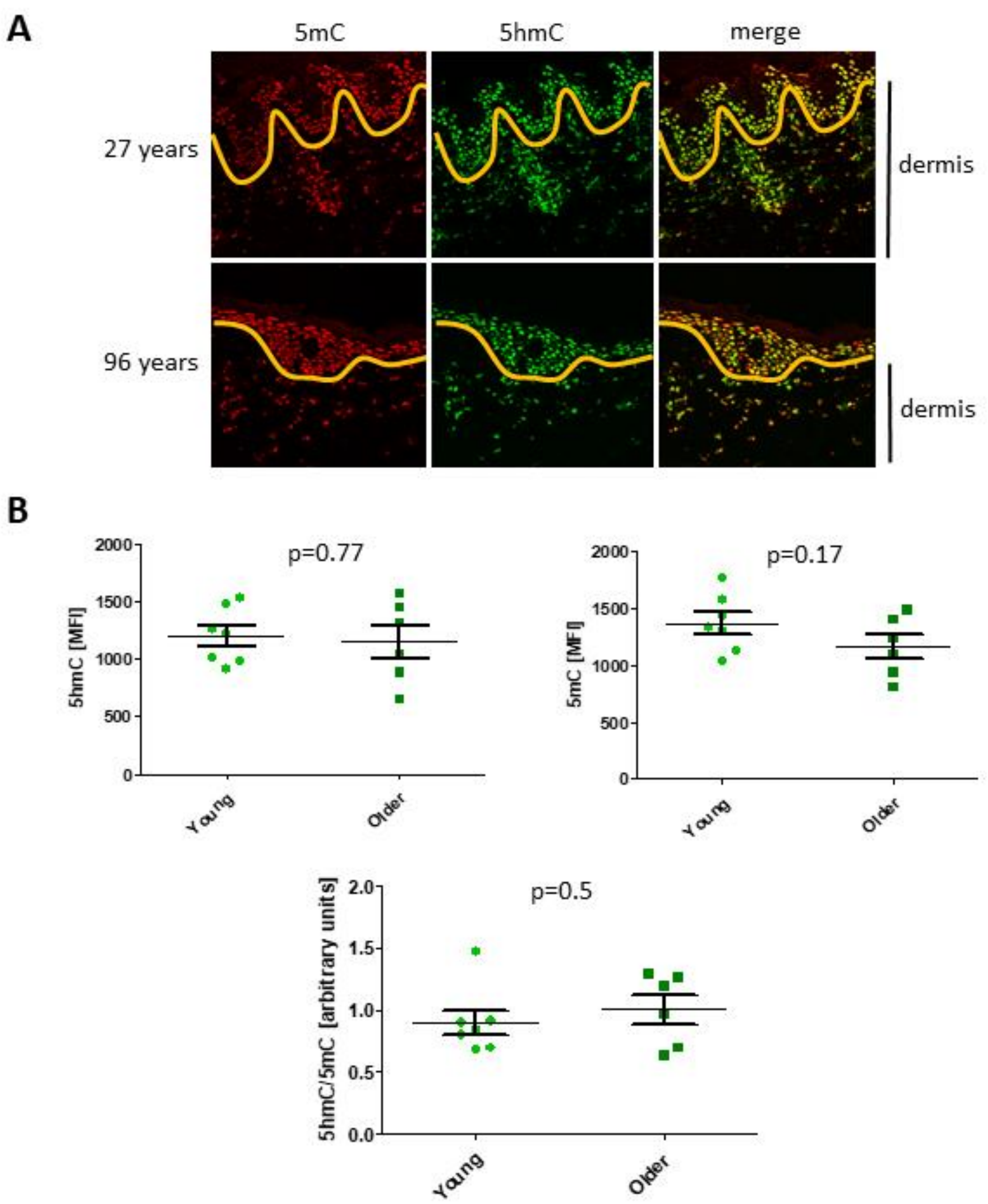

Supplementary Figure S2. A. Immunofluorescent detection of 5-methylcytosine (5mC) and 5-hydroxymethylcytosine (5hmC) in representative samples of whole-thickness skin of young (27 years) and age-advanced (96 years) individuals. Yellow lines divide dermis and epidermis. B. Results of the evaluation of the mean 5mC and 5hmC fluorescence intensity in dermal fibroblast nuclei using a confocal microscope and the ZEN 2012 version BLUE program. Each dot represents the mean fluorescence intensity of 100 nuclei in the dermis of a single study participant. The statistical analysis was performed with the Student's *t*-test. Young (22–35 years), older (74–94 years).

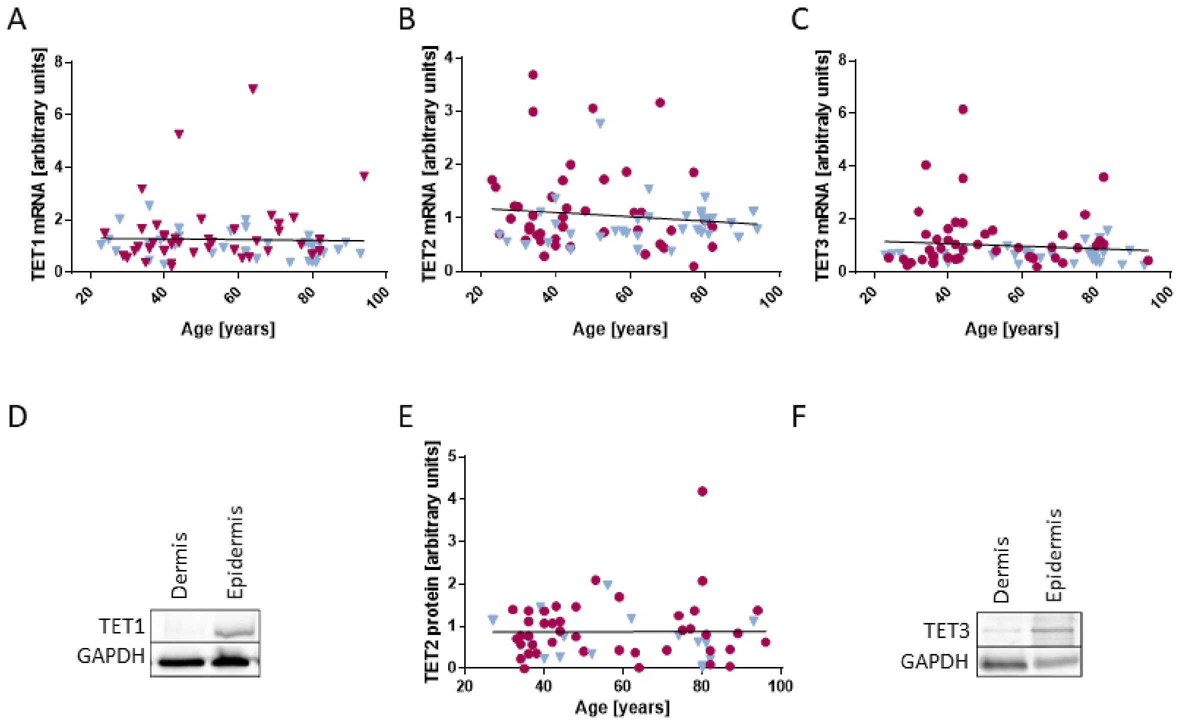

Supplementary Figure S3. Expression of TET1, TET2, and TET3 in the dermis of individuals of different ages. A., B., C. The expression of TET1, TET2, or TET3 mRNAs does not depend on age. The statistical analysis was performed with Spearman's correlation test. D. Immunoblot showing a lack of specific TET1 signal in the dermis. E. The expression of TET2 protein does not depend on age. The panel also shows an example of an immunoblot. F. Immunoblot showing a very weak specific TET3 signal in the dermis sample. In the majority of other samples, the TET3 signal was absent. The statistical analysis was performed with Spearman's correlation test. Purple circles: women. Blue triangles: men. Epidermis: positive control. GAPDH: internal loading control.

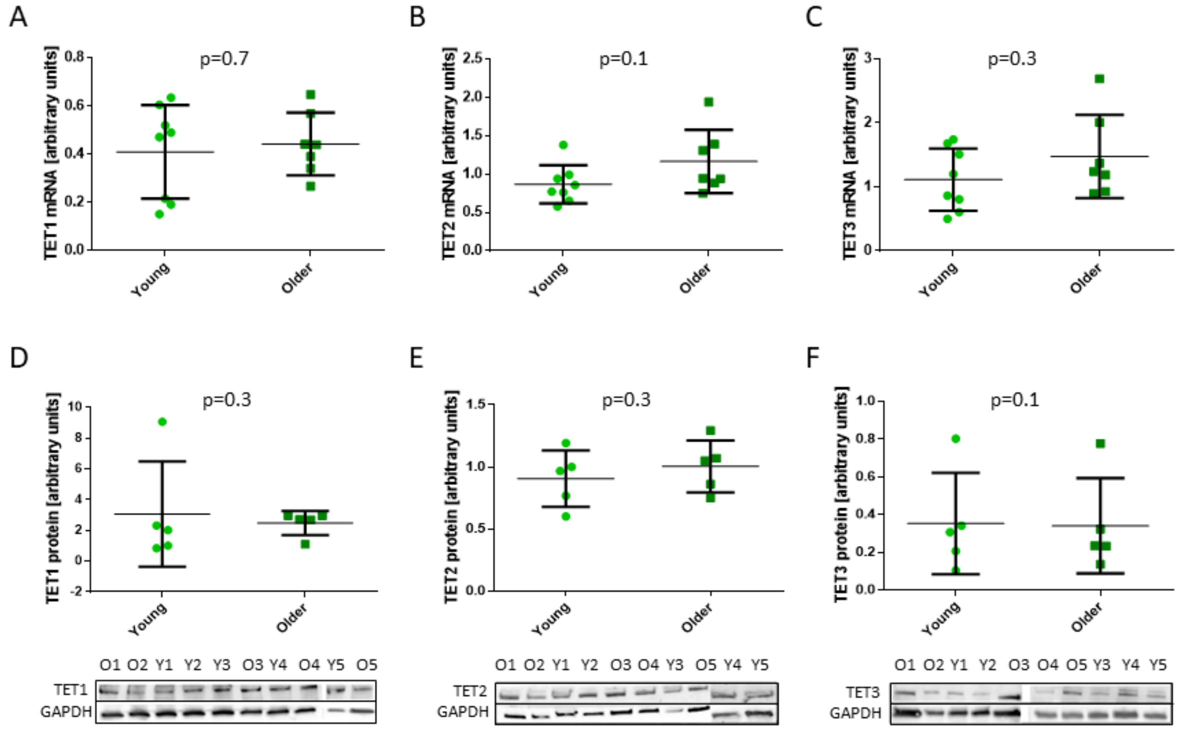

Supplementary Figure S4. Expression of TET1, TET2, and TET3 in primary fibroblasts. A., B., C. The expression of TET1, TET2, and TET3 mRNAs is similar in fibroblasts originating from young (22–35 years) and older (74–94 years) individuals. The statistical analysis was performed with the Student's *t*-test. D., E., F. The expression of TET1, TET2, and TET3 proteins does not differ between fibroblasts originating from young (22–35 years) and older (74–94 years) individuals. Panels D., E., F. also show examples of immunoblots. The statistical analysis was performed with the Mann-Whitney *U*-test. Y: younger. O: older. GAPDH: internal loading control.

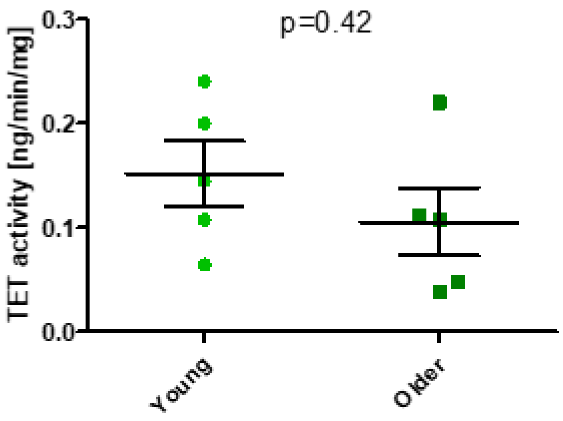

Supplementary Figure S5. TET proteins activity in primary fibroblasts originating from young (22–35 years) and older (74–94 years) individuals. The statistical analysis was performed with the Mann-Whitney *U*-test.

Supplementary Table S1. Age-related DHMRs in human dermal fibroblasts.

MEDIPS wide window

Chr	start	end	width	log2fold	adj. *p*-value	annotation	associated genes
**chr4**	9350551	9351450	900	6,44	8,73E-02	Distal Intergenic	USP17L28,USP17L27
**chr3**	63264601	63265050	450	3,58	3,05E-02	Intron	SYNPR
**chr1**	25283701	25285050	1350	3,51	5,39E-04	Exon	RSRP1,RHD
**chr8**	106508701	106509150	450	3,49	7,72E-02	Intron	OXR1
**chr1**	25285951	25287300	1350	3,45	4,78E-05	Intron	RSRP1,RHD
**chr13**	98364601	98365050	450	3,44	7,36E-02	Intron	FARP1
**chr3**	169557301	169557750	450	3,37	2,95E-02	Intron	MECOM
**chr18**	52973551	52974000	450	3,33	7,97E-02	Intron	DCC
**chr10**	89711101	89711550	450	3,25	9,35E-02	Exon	KIF20B
**chr2**	200465551	200466000	450	3,21	3,19E-02	Intron	SPATS2L
**chr1**	25274701	25276050	1350	3,17	3,48E-02	Intron	RSRP1,RHD
**chr1**	25329601	25330050	450	3,16	5,10E-02	3' UTR	RSRP1,RHD
**chr1**	25331851	25332300	450	3,14	5,90E-05	Intron	RSRP1,RHD
**chr1**	25312501	25312950	450	3,06	1,79E-02	Intron	RSRP1,RHD
**chr12**	13463551	13464000	450	3,05	7,97E-02	Intron	GRIN2B
**chr2**	30404701	30405150	450	3,02	5,32E-02	Distal Intergenic	LCLAT1
**chr1**	25321051	25321500	450	2,83	7,55E-02	Intron	RSRP1,RHD

**chr1**	57972151	57972600	450	2,81	9,80E-02	Intron	DAB1
**chr3**	36344701	36345150	450	2,81	8,76E-02	Distal Intergenic	STAC
**chr9**	118828351	118828800	450	2,81	6,46E-02	Distal Intergenic	LINC02578
**chr1**	25318801	25319250	450	2,81	7,15E-02	Intron	RSRP1,RHD
**chr15**	77196151	77196600	450	2,80	4,84E-02	Intron	PEAK1
**chr11**	55570051	55570500	450	2,77	4,64E-02	Promoter	OR4C16
**chr3**	9428851	9429300	450	2,75	6,74E-02	5' UTR	SETD5
**chr2**	49888801	49889250	450	2,71	7,36E-02	Exon	FSHR
**chr1**	16425451	16425900	450	2,54	8,59E-02	5' UTR	SPATA21
**chr4**	78406201	78406650	450	2,54	9,80E-02	Intron	FRAS1
**chr15**	45773101	45773550	450	2,46	9,80E-02	Intron	LOC105370802
**chr1**	25304851	25305300	450	2,38	1,52E-02	Intron	RSRP1,RHD
**chr1**	21123901	21124350	450	2,30	7,21E-02	Intron	EIF4G3
**chr7**	48560401	48560850	450	2,30	9,08E-02	Intron	ABCA13
**chr18**	48523501	48523950	450	2,28	9,80E-02	Distal Intergenic	CTIF
**chr6**	163437751	163438200	450	2,12	9,46E-02	Intron	QKI
**chr1**	25302601	25303050	450	2,12	7,91E-02	Intron	RSRP1,RHD
**chr1**	25292251	25293600	1350	2,10	1,38E-06	Intron	RSRP1,RHD
**chr5**	100171351	100171800	450	2,09	7,97E-02	Distal Intergenic	LOC100133050
**chr1**	221013451	221013900	450	2,02	8,76E-02	Distal Intergenic	HLX-AS1
**chr2**	41746051	41746500	450	2,01	8,76E-02	Distal Intergenic	LINC01913
**chr2**	29657251	29657700	450	1,91	9,38E-02	Intron	ALK
**chr14**	101169001	101169450	450	1,82	4,84E-02	Distal Intergenic	LINC02285
**chr1**	166662901	166663350	450	1,73	4,96E-02	Distal Intergenic	FMO9P
**chr16**	81595801	81596250	450	1,72	2,30E-02	Intron	CMIP
**chr19**	33504301	33504750	450	1,66	4,84E-02	Intron	PEPD
**chr5**	163507951	163508400	450	1,66	2,73E-02	Intron	MAT2B
**chr5**	119098801	119099250	450	1,66	9,74E-02	Intron	DMXL1
**chr10**	1860751	1861200	450	1,65	4,84E-02	Distal Intergenic	ADARB2
**chr21**	19431901	19432800	900	1,65	4,02E-02	Distal Intergenic	-
**chr14**	88584751	88585200	450	1,64	8,76E-02	Intron	ZC3H14
**chr2**	167633551	167634000	450	1,64	7,97E-02	Distal Intergenic	LOC105616981
**chr3**	24750001	24750450	450	1,62	4,84E-02	Intron	RARB
**chr19**	1629901	1630350	450	1,60	7,36E-02	Intron	TCF3
**chr4**	150087601	150088050	450	1,60	5,32E-02	Intron	DCLK2
**chr19**	11009251	11009700	450	1,59	2,73E-02	Intron	SMARCA4
**chr4**	75555001	75555450	450	1,57	6,48E-02	Promoter	ODAPH,THAP6
**chr1**	2186101	2186550	450	1,54	9,74E-02	3' UTR	FAAP20,PRKCZ-AS1,PRKCZ
**chr3**	114513751	114514200	450	1,54	7,91E-02	Intron	ZBTB20-AS5,ZBTB20
**chr5**	67573351	67573800	450	1,54	8,76E-02	Intron	LOC101928858
**chr15**	47640601	47641050	450	1,47	8,76E-02	Intron	SEMA6D
**chr18**	72913501	72913950	450	1,46	8,76E-02	Distal Intergenic	LOC100505797
**chr8**	25399351	25399800	450	1,41	7,91E-02	Intron	DOCK5
**chr7**	278101	278550	450	1,40	9,38E-02	Distal Intergenic	LOC442497

**chr16**	953551	954000	450	1,40	8,76E-02	Intron	LMF1
**chr1**	246796651	246797100	450	1,38	9,08E-02	Intron	LINC01341
**chr18**	12957751	12958200	450	1,38	7,97E-02	Intron	SEH1L
**chr2**	199820701	199821600	900	1,37	8,11E-02	Intron	FTCDNL1
**chr14**	96745951	96746850	900	1,37	4,03E-02	Intron	LINC02299
**chr3**	49672351	49672800	450	1,30	7,55E-02	Promoter	APEH,BSN
**chr13**	32597551	32598000	450	1,28	4,84E-02	Intron	PDS5B
**chr4**	62645851	62646300	450	1,28	3,05E-02	Distal Intergenic	ADGRL3-AS1
**chr10**	30328651	30329100	450	1,27	1,50E-02	Intron	MTPAP
**chr21**	36909901	36910350	450	1,24	7,21E-02	Intron	HLCS
**chr7**	144190801	144191250	450	1,23	2,87E-03	Intron	ARHGEF35,LOC101928605
**chr17**	19097551	19098000	450	1,20	7,99E-02	Distal Intergenic	SNORD3D
**chr1**	64974601	64975050	450	1,18	7,97E-02	Exon	LINC01359,JAK1
**chr8**	62874001	62874450	450	1,13	9,74E-02	Intron	NKAIN3
**chr14**	63229951	63230400	450	1,12	8,22E-03	Intron	RHOJ
**chr7**	144354601	144355050	450	1,11	4,84E-02	Promoter	ARHGEF5
**chr4**	37584901	37585800	900	1,03	4,60E-02	Intron	C4orf19,RELL1
**chr21**	8454601	8455050	450	0,96	5,32E-02	Distal Intergenic	FP236383.3-202
**chr2**	26517601	26518050	450	0,90	8,76E-02	Intron	OTOF
**chr14**	105055201	105055650	450	0,82	4,84E-02	5' UTR	GPR132
**chr17**	61402501	61403400	900	0,75	6,43E-02	3' UTR	TBX2-AS1,TBX2
**chr16**	16233301	16233750	450	0,71	8,76E-02	Intron	MIR3179-4
**chr12**	59328001	59328450	450	-0,45	5,45E-02	Distal Intergenic	SLC16A7
**chr3**	196099651	196100100	450	-0,54	8,37E-04	Distal Intergenic	TFRC
**chrX**	19543501	19543950	450	-0,64	2,73E-02	Intron	SH3KBP1
**chrX**	73080451	73080900	450	-0,73	3,07E-02	Promoter	PABPC1L2A
**chr6**	909001	909450	450	-0,77	3,80E-02	Exon	LINC01622
**chr14**	18656101	18656550	450	-0,87	5,32E-02	Distal Intergenic	LINC02297
**chr18**	76618801	76619250	450	-0,91	7,72E-02	Intron	LINC00683,LINC01927
**chr1**	6102901	6103350	450	-0,92	5,32E-02	3' UTR	CHD5,KCNAB2
**chr6**	909451	909900	450	-1,00	9,37E-02	Distal Intergenic	LINC01622
**chr1**	120267901	120268350	450	-1,03	6,54E-02	Intron	SEC22B
**chrX**	29977201	29977650	450	-1,08	4,94E-02	Distal Intergenic	MAGEB2
**chr1**	16780501	16780950	450	-1,15	6,46E-02	Intron	CROCC
**chr7**	158915701	158916150	450	-1,15	9,35E-02	Intron	WDR60
**chrX**	102436201	102436650	450	-1,15	3,66E-02	Intron	TMSB15A
**chrX**	72836101	72836550	450	-1,16	7,72E-02	Intron	FAM226A
**chrX**	3132451	3132900	450	-1,19	4,71E-02	Distal Intergenic	ARSF
**chr15**	20344951	20345400	450	-1,19	7,97E-02	Exon	CHEK2P2
**chrX**	50046301	50046750	450	-1,21	4,84E-02	Intron	CLCN5
**chrX**	85929301	85929750	450	-1,21	4,84E-02	Intron	CHM
**chrX**	74291851	74292300	450	-1,25	7,55E-02	Promoter	FTX,MIR374A,MIR545
**chr3**	89588701	89589150	450	-1,25	9,80E-02	Exon	EPHA3
**chrX**	50815351	50815800	450	-1,27	4,84E-02	Promoter	SHROOM4
**chrX**	85928851	85929300	450	-1,27	2,52E-02	Intron	CHM

**chr5**	3018601	3019050	450	-1,27	2,73E-02	Distal Intergenic	LINC01377
**chr12**	90738001	90738450	450	-1,33	7,72E-02	Distal Intergenic	LINC00615
**chr7**	14281201	14281650	450	-1,36	7,55E-02	Intron	DGKB
**chrX**	110414251	110414700	450	-1,36	6,46E-02	Intron	RTL9,AMMECR1
**chr5**	1626751	1627200	450	-1,36	7,63E-02	Exon	LOC728613
**chrX**	7378201	7378650	450	-1,38	3,74E-02	Distal Intergenic	STS
**chr12**	53187301	53187750	450	-1,43	6,54E-02	3' UTR	ZNF740,ITGB7
**chr6**	166784401	166784850	450	-1,44	5,32E-02	Intron	RPS6KA2
**chr7**	147549151	147549600	450	-1,44	9,08E-02	Intron	LOC105375556
**chr7**	14281651	14282100	450	-1,45	9,35E-02	Intron	DGKB
**chrX**	64487251	64487700	450	-1,46	7,63E-02	Distal Intergenic	MTMR8
**chrX**	48262951	48263400	450	-1,47	7,95E-02	Intron	SSX1
**chrX**	89827201	89827650	450	-1,47	7,72E-02	Distal Intergenic	TGIF2LX
**chr7**	111382201	111382650	450	-1,56	1,55E-02	Intron	IMMP2L
**chr15**	22141351	22141800	450	-1,59	1,67E-04	Distal Intergenic	LOC642131
**chrX**	3686401	3686850	450	-1,62	6,19E-02	Intron	PRKX
**chr15**	22140901	22141350	450	-1,66	1,47E-03	Distal Intergenic	LOC642131
**chrX**	149887351	149887800	450	-1,68	3,05E-02	Distal Intergenic	MAGEA8
**chrX**	101057401	101057850	450	-1,74	4,84E-02	Intron	TRMT2B
**chr7**	158916151	158916600	450	-1,74	2,30E-02	Intron	WDR60
**chr3**	174603601	174604050	450	-1,74	9,80E-02	Intron	NAALADL2
**chr10**	89527051	89527500	450	-1,80	7,55E-02	Intron	SLC16A12
**chr13**	69157801	69158250	450	-1,85	8,76E-02	Distal Intergenic	LINC00383
**chr22**	11922301	11922750	450	-1,89	5,16E-02	Distal Intergenic	FRG1FP
**chr6**	51735151	51735600	450	-2,01	8,32E-02	Intron	PKHD1
**chrX**	137206351	137206800	450	-2,09	2,73E-02	Distal Intergenic	GPR101
**chr6**	76407751	76408200	450	-2,12	9,18E-02	Distal Intergenic	LINC02540
**chrX**	88240501	88240950	450	-2,13	5,82E-02	Distal Intergenic	CPXCR1
**chr1**	25406101	25406550	450	-2,19	6,50E-02	Intron	RHCE
**chr22**	20587951	20588400	450	-2,20	9,74E-02	Exon	MED15
**chr1**	25405651	25406100	450	-2,24	2,30E-02	Intron	RHCE
**chr9**	35289901	35290350	450	-2,30	3,45E-02	Intron	UNC13B
**chr6**	4642201	4642650	450	-2,35	6,65E-02	Distal Intergenic	KU-MEL-3
**chr3**	180219601	180220050	450	-2,40	9,08E-02	Distal Intergenic	PEX5L
**chrX**	89767801	89768250	450	-2,47	2,30E-02	Distal Intergenic	TGIF2LX
**chr4**	6650101	6650550	450	-2,49	7,74E-02	Intron	LINC02482
**chr6**	51735601	51736050	450	-2,51	2,30E-02	Intron	PKHD1
**chr13**	68024251	68024700	450	-2,52	8,42E-02	Distal Intergenic	LINC00364
**chr13**	69468751	69469200	450	-2,53	1,40E-02	Distal Intergenic	LINC00383
**chrX**	51088501	51088950	450	-2,66	7,99E-02	Exon	LINC01284
**chr11**	10000801	10001250	450	-2,75	8,97E-02	Exon	SBF2
**chrX**	147693151	147693600	450	-2,80	5,80E-03	Distal Intergenic	FMR1
**chrX**	119997451	119997900	450	-2,93	7,91E-02	Intron	RHOXF1P1
**chr14**	106757551	106758000	450	-2,95	6,74E-02	Distal Intergenic	MIR5195
**chr11**	55616851	55617300	450	-3,03	7,00E-02	Distal Intergenic	OR4C11

**chrX**	6764401	6764850	450	-3,18	8,97E-02	Distal Intergenic	VCX3A
**chr2**	115168501	115168950	450	-3,31	2,74E-03	Intron	DPP10
**chrX**	88698151	88698600	450	-3,99	7,36E-02	Distal Intergenic	CPXCR1
**chr6**	117029701	117030150	450	-4,24	3,59E-02	Distal Intergenic	RFX6
**chr15**	68535451	68535900	450	-4,24	8,76E-02	Distal Intergenic	CORO2B
**chr1**	71516251	71516700	450	-4,29	6,05E-02	Intron	NEGR1
**chr4**	44492401	44492850	450	-4,29	7,55E-02	Distal Intergenic	KCTD8
**chr1**	71515801	71516250	450	-4,34	4,84E-02	Intron	NEGR1
**chrX**	82856701	82857150	450	-5,18	8,59E-02	Distal Intergenic	POU3F4
**chrX**	112947751	112948200	450	-5,42	7,55E-02	Distal Intergenic	AMOT

MEDIPS narrow window

Chr	start	end	width	log2fold	adj. p-value	annotation	associated genes
**chr4**	9351001	9351100	100	6,63	9,19E-02	Distal Intergenic	USP17L28
**chr1**	25312701	25312750	50	4,32	9,19E-03	Intron	RSRP1,RHD
**chr4**	65423251	65423300	50	4,27	9,19E-03	Intron	EPHA5
**chr1**	25332101	25332200	100	4,00	1,99E-06	Intron	RSRP1,RHD
**chr2**	200465801	200465900	100	3,97	7,16E-02	Intron	SPATS2L
**chr1**	25330051	25330150	100	3,93	2,84E-02	3' UTR	RSRP1,RHD
**chr1**	25283951	25284000	50	3,91	9,75E-02	Intron	RSRP1,RHD
**chr16**	83481401	83481450	50	3,91	9,61E-02	Intron	CDH13
**chr11**	3178201	3178250	50	3,91	1,17E-03	Distal Intergenic	OSBPL5
**chr1**	25286501	25286900	400	3,73	1,08E-07	Intron	RSRP1,RHD
**chr1**	25284801	25284850	50	3,47	2,02E-02	Intron	RSRP1,RHD
**chr1**	25284451	25284750	300	3,46	7,39E-03	Exon	RSRP1,RHD
**chr1**	25284201	25284250	50	3,38	1,18E-03	Intron	RSRP1,RHD
**chr2**	40817001	40817050	50	3,29	2,68E-02	Distal Intergenic	SLC8A1
**chr1**	25284351	25284400	50	3,28	4,86E-03	Intron	RSRP1,RHD
**chr1**	25293401	25293450	50	3,27	3,31E-05	Intron	RSRP1,RHD
**chr1**	25286401	25286450	50	3,24	1,25E-03	Intron	RSRP1,RHD
**chr1**	25332251	25332300	50	3,23	1,55E-02	Intron	RSRP1,RHD
**chr1**	25274701	25274950	250	3,18	1,24E-02	Intron	RSRP1,RHD
**chr3**	141814001	141814050	50	3,16	6,37E-02	Intron	GRK7
**chr1**	25286301	25286350	50	3,16	9,89E-03	Intron	RSRP1,RHD
**chr12**	132725201	132725250	50	3,16	8,58E-02	Distal Intergenic	PGAM5,ANKLE2
**chr1**	25275051	25275200	150	3,12	2,58E-03	Intron	RSRP1,RHD
**chr1**	25331801	25332050	250	3,11	6,61E-03	Intron	RSRP1,RHD
**chr4**	151972201	151972250	50	3,07	9,28E-02	Distal Intergenic	LINC02273
**chr1**	25286951	25287100	150	3,02	1,16E-04	Intron	RSRP1,RHD
**chr1**	25275301	25275400	100	2,98	3,91E-02	Intron	RSRP1,RHD
**chr1**	25287151	25287200	50	2,89	1,17E-02	Intron	RSRP1,RHD
**chr14**	103814051	103814100	50	2,89	6,00E-02	Intron	PPP1R13B

**chr18**	23420951	23421000	50	2,73	9,61E-02	Intron	TMEM241
**chr1**	25293301	25293350	50	2,73	7,11E-03	Intron	RSRP1,RHD
**chrX**	23227151	23227250	100	2,69	9,01E-02	Intron	PTCHD1-AS
**chr1**	25318701	25318750	50	2,66	9,07E-02	Intron	RSRP1,RHD
**chr2**	41746101	41746150	50	2,61	3,52E-02	Distal Intergenic	LINC01913
**chr10**	6158001	6158100	100	2,51	4,16E-02	Intron	PFKFB3
**chr3**	159668751	159668800	50	2,49	9,75E-02	Intron	IQCJ-SCHIP1,SCHIP1
**chr2**	234448551	234448600	50	2,44	6,30E-02	Intron	LINC01891
**chr19**	51792651	51792700	50	2,35	7,37E-02	Intron	FPR1,FPR3
**chr7**	144247851	144247900	50	2,34	3,06E-02	Intron	LOC101928605
**chr1**	25293201	25293250	50	2,33	3,21E-05	Intron	RSRP1,RHD
**chr13**	51457351	51457400	50	2,32	7,96E-02	Intron	INTS6-AS1,INTS6
**chr13**	51457601	51457650	50	2,30	7,16E-02	Intron	INTS6-AS1,INTS6
**chr16**	54979551	54979600	50	2,30	7,55E-02	Distal Intergenic	IRX5
**chr12**	85672001	85672050	50	2,30	5,91E-02	Distal Intergenic	RASSF9
**chr9**	67578951	67579050	100	2,28	7,12E-02	Distal Intergenic	CBWD5
**chr1**	25305701	25305850	150	2,27	6,19E-02	Intron	RSRP1,RHD
**chr17**	43056251	43056300	50	2,26	7,35E-02	Intron	BRCA1
**chr9**	77794501	77794550	50	2,22	2,85E-02	Exon	GNAQ
**chr14**	83529651	83529750	100	2,13	3,08E-02	Distal Intergenic	LINC02301
**chr19**	33504651	33504750	100	2,11	2,43E-02	Intron	PEPD
**chr8**	138943551	138943600	50	2,10	6,26E-02	Distal Intergenic	COL22A1
**chr14**	63229951	63230000	50	2,08	7,26E-02	Intron	RHOJ
**chr3**	108391401	108391450	50	2,08	9,06E-02	Intron	MYH15
**chr1**	25292351	25292400	50	2,05	1,11E-02	Intron	RSRP1,RHD
**chr19**	11009301	11009350	50	2,05	8,88E-02	Intron	SMARCA4
**chr1**	25292451	25292550	100	2,04	2,57E-03	Intron	RSRP1,RHD
**chr11**	115697851	115697900	50	2,03	3,82E-02	Intron	LINC00900
**chr1**	25292601	25292650	50	2,03	2,60E-06	Intron	RSRP1,RHD
**chr14**	81590651	81590700	50	2,02	9,07E-02	Distal Intergenic	LINC01467
**chr1**	25292951	25293050	100	2,02	1,27E-06	Intron	RSRP1,RHD
**chr2**	109845501	109845550	50	1,97	7,53E-02	Intron	RGPD6
**chr1**	153580251	153580300	50	1,97	9,61E-02	Distal Intergenic	S100A2
**chr1**	25295601	25295650	50	1,96	7,03E-02	Intron	RSRP1,RHD
**chr1**	232947251	232947300	50	1,94	7,69E-02	Distal Intergenic	NTPCR
**chr12**	4181801	4182000	200	1,88	6,05E-02	Distal Intergenic	TIGAR
**chr6**	76219501	76219550	50	1,87	6,97E-02	Distal Intergenic	IMPG1
**chr4**	28588601	28588650	50	1,87	1,99E-02	Intron	MIR4275
**chr17**	71545501	71545550	50	1,86	7,53E-02	Distal Intergenic	CASC17
**chr16**	81596001	81596050	50	1,86	3,81E-02	Intron	CMIP
**chr16**	81596101	81596150	50	1,85	9,75E-02	Intron	CMIP
**chr21**	19432301	19432350	50	1,83	2,86E-02	Distal Intergenic	-
**chr1**	166663301	166663350	50	1,81	8,25E-02	Distal Intergenic	FMO9P
**chr2**	26138151	26138200	50	1,81	4,91E-02	Distal Intergenic	GAREM2,RAB10
**chr22**	18981701	18981750	50	1,79	9,87E-02	Intron	DGCR5

**chr3**	128694551	128694750	200	1,79	4,70E-02	Distal Intergenic	RPN1
**chr2**	135981401	135981450	50	1,75	6,96E-02	Intron	DARS,DARS-AS1
**chr16**	81596201	81596250	50	1,74	9,67E-02	Intron	CMIP
**chr14**	63230151	63230200	50	1,70	3,73E-02	Intron	RHOJ
**chr14**	96746601	96746650	50	1,67	2,86E-02	Intron	LINC02299
**chr2**	26138001	26138050	50	1,66	9,75E-02	Distal Intergenic	GAREM2,RAB10
**chr12**	124433251	124433300	50	1,65	7,53E-02	Intron	NCOR2
**chr10**	1860951	1861050	100	1,65	6,38E-02	Distal Intergenic	ADARB2
**chr8**	25399601	25399650	50	1,63	2,35E-02	Intron	DOCK5
**chr14**	96746351	96746400	50	1,63	9,75E-02	Intron	LINC02299
**chr8**	133890051	133890100	50	1,63	8,84E-02	Intron	LOC101927822
**chr2**	2745801	2745850	50	1,63	7,73E-02	Distal Intergenic	LINC01250
**chr2**	2746001	2746050	50	1,62	9,67E-02	Distal Intergenic	LINC01250
**chr19**	45246051	45246100	50	1,61	7,69E-02	Intron	MARK4,EXOC3L2
**chr21**	15133551	15133600	50	1,60	6,97E-02	Distal Intergenic	AF222684.1-201
**chr1**	92611651	92611700	50	1,60	6,03E-02	Intron	EVI5
**chr2**	227184901	227185000	100	1,59	5,22E-02	Intron	COL4A3
**chr13**	32597801	32597850	50	1,59	4,16E-02	Intron	PDS5B
**chr1**	156716951	156717050	100	1,58	8,84E-02	Distal Intergenic	CRABP2,ISG20L2
**chr21**	8463601	8463650	50	1,54	6,29E-02	Distal Intergenic	-
**chr2**	199821151	199821300	150	1,53	5,11E-02	Intron	FTCDNL1
**chr11**	49295851	49295900	50	1,49	6,26E-02	Distal Intergenic	FOLH1
**chr15**	58873951	58874000	50	1,43	9,49E-02	Intron	RNF111
**chr19**	53223851	53223900	50	1,41	9,48E-02	Distal Intergenic	ZNF665
**chr6**	11709501	11709550	50	1,37	9,19E-02	Distal Intergenic	ADTRP
**chr1**	15675101	15675150	50	1,37	3,35E-02	Distal Intergenic	PLEKHM2
**chr17**	19097751	19097800	50	1,36	4,32E-02	Distal Intergenic	SNORD3D
**chr3**	72022401	72022450	50	1,35	2,92E-02	Distal Intergenic	LINC00870
**chr14**	49568901	49568950	50	1,35	9,75E-02	Distal Intergenic	RN7SL1,RPS29
**chr7**	144191001	144191150	150	1,35	4,47E-03	Intron	ARHGEF35,LOC101928605
**chr20**	56560501	56560550	50	1,31	7,41E-02	Distal Intergenic	LINC01716
**chr19**	366301	366450	150	1,31	7,76E-03	Intron	THEG
**chr19**	47196001	47196100	100	1,27	5,84E-02	Intron	SAE1
**chr12**	64483951	64484000	50	1,24	2,52E-02	Intron	TBK1
**chr10**	30328801	30328850	50	1,24	4,55E-02	Intron	MTPAP
**chr4**	37585101	37585150	50	1,20	4,72E-02	Intron	C4orf19
**chr12**	3075101	3075150	50	1,20	9,56E-02	Promoter	TSPAN9
**chr2**	49123001	49123050	50	1,17	4,25E-02	Intron	FSHR
**chr1**	15675201	15675250	50	1,16	2,02E-02	Distal Intergenic	PLEKHM2
**chr13**	50172251	50172300	50	1,15	7,42E-02	Exon	DLEU1,ST13P4
**chr19**	18913001	18913050	50	1,07	9,31E-02	3' UTR	COPE,HOMER3
**chr2**	26517901	26517950	50	1,01	1,45E-02	Intron	OTOF
**chr14**	105055501	105055650	150	0,87	4,78E-02	5' UTR	GPR132
**chr21**	8454551	8454600	50	0,81	9,56E-02	Distal Intergenic	FP236383.3-202
**chr16**	16233251	16233350	100	0,79	5,23E-02	Intron	MIR3179-4

**chr17**	61402901	61402950	50	0,77	7,32E-02	Intron	TBX2-AS1,TBX2
**chr19**	54055101	54055150	50	0,59	6,29E-02	Intron	CACNG6
**chr3**	196099751	196099850	100	-0,56	1,57E-02	Distal Intergenic	TFRC
**chr12**	59328401	59328450	50	-0,65	4,24E-02	Distal Intergenic	SLC16A7
**chr15**	75783151	75783200	50	-0,65	9,75E-02	Exon	MIR4313
**chr12**	448751	448800	50	-0,66	5,91E-02	Distal Intergenic	B4GALNT3
**chr20**	47098601	47098650	50	-0,67	9,07E-02	Intron	EYA2
**chrX**	73080851	73080900	50	-0,77	2,68E-02	Promoter	PABPC1L2A
**chr6**	909151	909300	150	-0,77	1,27E-02	Distal Intergenic	LINC01622
**chr19**	50249551	50249600	50	-0,85	3,82E-02	Intron	MYH14
**chrX**	71541551	71541600	50	-0,86	6,92E-02	Intron	OGT
**chrX**	73080651	73080750	100	-0,87	3,86E-02	Promoter	PABPC1L2A
**chr12**	72758251	72758300	50	-0,91	8,06E-02	Distal Intergenic	LINC02444
**chr1**	6103001	6103100	100	-0,91	6,43E-02	3' UTR	CHD5,KCNAB2
**chr18**	76618801	76618850	50	-0,91	6,26E-02	Intron	LINC00683,LINC01927
**chrX**	13050701	13050750	50	-0,92	6,97E-02	Distal Intergenic	FAM9C
**chr17**	505101	505150	50	-0,94	9,42E-02	Intron	RFLNB,VPS53
**chr6**	909351	909600	250	-0,95	6,20E-02	Distal Intergenic	LINC01622
**chr4**	1144451	1144600	150	-0,98	1,64E-02	Intron	TMED11P
**chr7**	158915751	158915800	50	-1,03	9,75E-02	Intron	WDR60
**chrX**	55069801	55069900	100	-1,06	8,01E-02	Distal Intergenic	PAGE2B
**chr3**	196099551	196099650	100	-1,06	4,58E-02	Distal Intergenic	TFRC
**chr1**	34019751	34019800	50	-1,13	9,61E-02	Intron	CSMD2
**chr1**	6103151	6103200	50	-1,13	3,65E-02	3' UTR	CHD5,KCNAB2
**chr1**	16780401	16780450	50	-1,13	7,53E-02	Intron	CROCC
**chr9**	129641201	129641250	50	-1,17	4,72E-02	Promoter	ASB6
**chr7**	45084401	45084450	50	-1,18	8,25E-02	Exon	NACAD
**chrX**	102436301	102436350	50	-1,20	4,32E-02	Intron	TMSB15A
**chrX**	3132551	3132650	100	-1,20	5,52E-02	Distal Intergenic	ARSF
**chr2**	58573301	58573400	100	-1,22	6,88E-02	Intron	LINC01122
**chrX**	50815401	50815450	50	-1,22	8,81E-02	Promoter	SHROOM4
**chr9**	129060751	129060800	50	-1,23	9,49E-02	Intron	MIGA2
**chrX**	85929051	85929100	50	-1,24	8,70E-02	Intron	CHM
**chrX**	85929201	85929250	50	-1,30	3,04E-02	Intron	CHM
**chrX**	46379051	46379100	50	-1,33	3,82E-02	Distal Intergenic	LINC01186
**chrX**	53863851	53863900	50	-1,34	3,82E-02	Distal Intergenic	HUWE1
**chr1**	16780551	16780600	50	-1,35	1,45E-02	Intron	CROCC
**chrX**	46378901	46378950	50	-1,42	6,97E-02	Distal Intergenic	LINC01186
**chrX**	56130201	56130250	50	-1,47	6,03E-02	Distal Intergenic	KLF8
**chrX**	56130101	56130150	50	-1,47	3,95E-02	Distal Intergenic	KLF8
**chr7**	158915951	158916150	200	-1,52	5,81E-02	Intron	WDR60
**chr22**	11818351	11818400	50	-1,61	7,74E-02	Distal Intergenic	FRG1FP
**chr15**	22141251	22141550	300	-1,62	5,33E-03	Distal Intergenic	LOC642131
**chr14**	87888801	87888850	50	-1,63	4,32E-02	Intron	GALC
**chrX**	110414551	110414600	50	-1,64	4,86E-02	Intron	RTL9,AMMECR1

**chr6**	166784301	166784400	100	-1,64	3,72E-02	Intron	RPS6KA2
**chr1**	110528501	110528550	50	-1,65	7,73E-02	Intron	KCNA2
**chrX**	48263001	48263050	50	-1,68	7,96E-02	Intron	SSX1
**chr22**	11818451	11818500	50	-1,69	7,90E-02	Distal Intergenic	FRG1FP
**chr7**	147549301	147549350	50	-1,71	8,58E-02	Intron	LOC105375556
**chr7**	14281701	14281750	50	-1,72	3,27E-02	Intron	DGKB
**chrX**	149887551	149887600	50	-1,72	9,28E-02	Distal Intergenic	MAGEA8
**chrX**	32177551	32177600	50	-1,73	9,56E-02	Intron	DMD
**chr15**	22141151	22141200	50	-1,76	1,68E-02	Distal Intergenic	LOC642131
**chr14**	70791701	70791750	50	-1,76	4,24E-02	Intron	MAP3K9
**chr22**	11922551	11922600	50	-1,88	4,72E-02	Distal Intergenic	FRG1FP
**chr7**	14281501	14281550	50	-1,94	2,67E-03	Intron	DGKB
**chr7**	29355201	29355250	50	-2,02	7,83E-02	Intron	CHN2
**chrX**	150541201	150541250	50	-2,06	7,73E-02	Distal Intergenic	MTM1
**chr9**	76575301	76575350	50	-2,07	2,86E-02	Intron	GCNT1
**chrX**	6533751	6533850	100	-2,07	1,63E-02	Promoter	VCX3A
**chrX**	88240651	88240700	50	-2,08	4,25E-02	Distal Intergenic	CPXCR1
**chrX**	42943701	42943750	50	-2,18	3,25E-02	Distal Intergenic	PPP1R2C
**chr1**	25405901	25405950	50	-2,29	7,13E-03	Intron	RHCE
**chr9**	90970851	90970900	50	-2,30	4,55E-02	Distal Intergenic	LINC00484
**chr5**	107490451	107490500	50	-2,35	7,25E-02	Intron	EFNA5
**chr1**	25406201	25406450	250	-2,37	1,27E-02	Intron	RHCE
**chr12**	53705251	53705300	50	-2,38	4,77E-02	Distal Intergenic	CALCOCO1
**chr20**	18197651	18197700	50	-2,40	4,72E-02	Distal Intergenic	KAT14
**chr10**	4167301	4167400	100	-2,40	9,75E-02	Distal Intergenic	LINC00702
**chr12**	84754851	84754900	50	-2,41	7,73E-02	Distal Intergenic	SLC6A15
**chr1**	25406001	25406050	50	-2,48	8,44E-05	Intron	RHCE
**chr22**	15739051	15739100	50	-2,52	9,75E-02	Distal Intergenic	LINC01297
**chr10**	16663451	16663500	50	-2,52	9,75E-02	Intron	RSU1
**chr6**	51735501	51735550	50	-2,54	2,20E-02	Intron	PKHD1
**chr10**	89527201	89527300	100	-2,61	5,49E-02	Intron	SLC16A12
**chrX**	137206451	137206500	50	-2,62	3,26E-02	Distal Intergenic	GPR101
**chrX**	147693551	147693600	50	-2,66	9,67E-02	Distal Intergenic	FMR1-AS1
**chr10**	124292801	124292850	50	-2,70	2,02E-02	Distal Intergenic	OAT
**chrX**	137206651	137206700	50	-2,73	4,72E-02	Distal Intergenic	GPR101
**chr7**	158916351	158916700	350	-2,74	4,67E-02	Intron	WDR60
**chrX**	28448051	28448100	50	-2,83	5,91E-02	Distal Intergenic	MIR6134
**chr6**	51735601	51735650	50	-2,89	3,20E-03	Intron	PKHD1
**chr4**	6650151	6650200	50	-2,90	7,56E-03	Intron	LINC02482
**chr14**	71365351	71365400	50	-2,92	9,56E-02	Intron	SIPA1L1
**chr7**	2064251	2064300	50	-2,93	3,74E-02	Intron	MAD1L1
**chr9**	35289801	35289850	50	-3,05	5,30E-02	Intron	UNC13B
**chrX**	120251901	120251950	50	-3,09	9,28E-02	Promoter	ZBTB33
**chr20**	52615601	52615650	50	-3,09	9,28E-02	Intron	LINC01524
**chrX**	137206351	137206400	50	-3,11	3,44E-02	Distal Intergenic	GPR101

**chr9**	35289901	35289950	50	-3,12	2,48E-02	Intron	UNC13B
**chr12**	72362451	72362500	50	-3,27	7,53E-02	Intron	TRHDE
**chr14**	106101151	106101200	50	-3,60	1,95E-02	Distal Intergenic	LINC00226
**chrX**	83190601	83190650	50	-3,89	4,32E-02	Distal Intergenic	POU3F4
**chr3**	175051801	175051900	100	-3,92	6,16E-02	Intron	NAALADL2
**chr6**	57935601	57935650	50	-3,99	6,97E-02	Intron	LINC00680-GUSBP4
**chr4**	176689501	176689550	50	-3,99	6,97E-02	Exon	VEGFC
**chr1**	71516151	71516200	50	-4,15	9,61E-02	Intron	NEGR1
**chr14**	106097001	106097050	50	-4,16	7,92E-02	Distal Intergenic	LINC00226
**chr2**	115168651	115168750	100	-4,34	3,69E-03	Intron	DPP10
**chr11**	55617151	55617250	100	-4,81	2,17E-02	Distal Intergenic	OR4C11
**chr18**	45729201	45729250	50	-5,12	7,53E-02	5' UTR	SLC14A1
**chrX**	123058301	123058350	50	-5,12	7,53E-02	Distal Intergenic	GRIA3
**chrX**	82856801	82856850	50	-5,12	7,53E-02	Distal Intergenic	POU3F4
**chr13**	69469001	69469150	150	-5,16	5,78E-04	Distal Intergenic	LINC00383
**chrX**	123058201	123058250	50	-5,18	4,95E-02	Distal Intergenic	GRIA3
**chr9**	124658701	124658750	50	-5,37	8,20E-02	Promoter	MIR181A2HG,NR6A1
**chrX**	89767851	89767900	50	-5,47	3,45E-02	Distal Intergenic	TGIF2LX
**chr1**	228558351	228558400	50	-6,26	1,99E-02	Distal Intergenic	BTNL10

DiffBind wide window

**Chr**	**start**	**end**	**width**	**log2fold**	**adj. *p*-value**	**annotation**	**associated genes**
**chr1**	25292446	25293163	718	3,34	3,71E-03	Intron	RSRP1,RHD
**chr1**	25323259	25323603	345	2,82	6,16E-02	Intron	RSRP1,RHD
**chr16**	952540	954220	1681	2,62	9,14E-03	Exon	LMF1
**chr9**	61944523	61944932	410	2,46	3,95E-03	Intron	FAM27C
**chr15**	30182742	30183080	339	2,41	5,17E-05	Distal Intergenic	LINC02249
**chr9**	61867523	61868052	530	2,32	7,70E-03	Distal Intergenic	FAM27C
**chr11**	70345235	70345519	285	2,31	6,47E-02	Intron	PPFIA1
**chr2**	240680748	240681090	343	2,28	3,50E-02	Promoter	AQP12B
**chrX**	268926	270514	1589	2,28	4,80E-02	Distal Intergenic	XG
**chr2**	107906499	107906787	289	2,28	7,15E-02	Distal Intergenic	RGPD4
**chr21**	13368082	13368420	339	2,01	1,30E-02	Distal Intergenic	-
**chr9**	63776344	63776715	372	1,80	4,50E-02	Distal Intergenic	LOC286297
**chr7**	144236412	144236927	516	1,77	4,33E-02	Intron	LOC101928605
**chr3**	128694656	128695095	440	1,70	2,50E-02	Distal Intergenic	RPN1
**chr21**	8448539	8449728	1190	1,66	4,73E-02	Distal Intergenic	FP236383.3-202
**chr16**	88500924	88501785	862	1,64	6,81E-02	Intron	ZFPM1
**chr13**	18230159	18230726	568	1,60	5,74E-02	Exon	FAM230C
**chr15**	48529745	48530427	683	1,53	3,37E-02	Intron	FBN1
**chr20**	63234004	63234398	395	1,50	7,38E-03	Promoter	BIRC7,MIR3196
**chr10**	30328698	30329009	312	1,43	3,96E-03	Intron	MTPAP
**chr3**	103896348	103896693	346	1,43	3,50E-02	Distal Intergenic	MIR548AB

**chr10**	65524826	65525155	330	1,38	2,38E-02	Distal Intergenic	LINC01515
**chr16**	18286591	18286921	331	1,33	4,80E-02	Distal Intergenic	MIR6770-2
**chrX**	12193586	12193975	390	1,32	9,11E-02	Intron	FRMPD4
**chr6**	28237312	28237616	305	1,29	7,70E-03	Distal Intergenic	ZSCAN9
**chr8**	109762969	109763258	290	1,29	4,80E-02	Distal Intergenic	SYBU
**chr20**	56560647	56561031	385	1,28	7,70E-03	Distal Intergenic	LINC01716
**chr9**	62039183	62039604	422	1,26	7,70E-03	Intron	FAM27C
**chr16**	19934436	19934742	307	1,26	4,43E-02	Distal Intergenic	GPRC5B
**chr4**	15884899	15885193	295	1,24	5,93E-02	Distal Intergenic	FGFBP1
**chr1**	120465046	120465479	434	1,24	9,14E-02	Exon	NBPF8
**chr12**	64483872	64484249	378	1,22	7,70E-03	Exon	TBK1
**chr2**	39005664	39006012	349	1,21	3,09E-02	Intron	SOS1
**chr6**	149983781	149984605	825	1,18	5,07E-02	Distal Intergenic	ULBP1
**chr2**	128921270	128921584	315	1,18	6,16E-02	Distal Intergenic	LINC01854
**chr20**	23316554	23316854	301	1,17	2,34E-02	Distal Intergenic	NXT1
**chr9**	118140757	118141085	329	1,16	1,41E-02	Distal Intergenic	TLR4
**chr2**	7165126	7165427	302	1,16	4,03E-02	Distal Intergenic	LOC101929452
**chr7**	144363448	144363881	434	1,16	8,68E-02	Exon	ARHGEF5
**chr11**	109294490	109294799	310	1,15	9,97E-02	Distal Intergenic	C11orf87
**chr2**	219593845	219594219	375	1,14	4,05E-02	Distal Intergenic	STK11IP
**chr22**	42660813	42661116	304	1,14	7,74E-02	Distal Intergenic	CYB5R3
**chr9**	30181504	30181782	279	1,13	9,12E-02	Distal Intergenic	LINC01242
**chr5**	89033043	89033454	412	1,12	7,61E-02	Intron	MEF2C-AS1
**chr7**	3160844	3161143	300	1,12	7,74E-02	Intron	LOC100129603
**chr4**	127548091	127548474	384	1,10	1,63E-02	Distal Intergenic	INTU
**chr9**	341512	341785	274	1,10	5,93E-02	Intron	DOCK8
**chr8**	18155143	18155434	292	1,10	8,49E-02	Distal Intergenic	NAT1
**chr10**	109968563	109968873	311	1,09	3,00E-02	Intron	ADD3-AS1
**chr10**	61487775	61488056	282	1,08	7,38E-03	Distal Intergenic	TMEM26
**chr1**	181539087	181539408	322	1,08	9,65E-02	Intron	CACNA1E
**chr5**	15454662	15455001	340	1,07	7,65E-02	Distal Intergenic	FBXL7
**chr8**	63776758	63777174	417	1,07	7,77E-02	Intron	LINC01414,LINC01289
**chr1**	80236173	80236514	342	1,07	9,55E-02	Distal Intergenic	LINC01781
**chr16**	49399328	49399675	348	1,06	8,79E-02	3' UTR	C16orf78
**chr18**	3726226	3726623	398	1,05	7,40E-03	Intron	DLGAP1
**chr4**	23659029	23659346	318	1,05	7,71E-02	Intron	PPARGC1A
**chr1**	12369040	12369323	284	1,04	5,79E-02	Intron	VPS13D
**chr6**	36338257	36338622	366	1,04	8,35E-02	Promoter	C6orf222
**chr2**	143013079	143013446	368	1,03	5,68E-02	Intron	KYNU
**chr11**	113213884	113214287	404	1,03	5,93E-02	Intron	NCAM1
**chr2**	47563126	47563527	402	1,01	7,70E-03	3' UTR	MSH2,KCNK12
**chr4**	80165836	80166342	507	1,01	1,65E-02	Distal Intergenic	PRDM8
**chr2**	176676762	176677116	355	1,00	2,35E-02	Distal Intergenic	LINC01117
**chr9**	72892924	72893210	287	1,00	7,74E-02	Distal Intergenic	LINC01474
**chr2**	58367141	58367441	301	1,00	9,74E-02	Distal Intergenic	LINC01122

**chr1**	234964470	234964881	412	0,99	7,70E-03	Intron	LOC101927851
**chr16**	66852301	66852691	391	0,98	5,07E-02	Intron	CA7,NAE1
**chr7**	42051849	42052172	324	0,98	5,07E-02	Intron	GLI3
**chr7**	130820136	130820609	474	0,97	3,00E-02	Intron	LINC-PINT
**chr12**	62086978	62087251	274	0,97	7,65E-02	Intron	TAFA2
**chr7**	43064909	43065335	427	0,97	8,79E-02	Intron	HECW1
**chr6**	14376812	14377112	301	0,97	9,90E-02	Distal Intergenic	LINC01108
**chr5**	165869356	165869707	352	0,96	7,65E-02	Distal Intergenic	LINC01947
**chr8**	123875832	123876300	469	0,95	7,71E-02	Intron	FER1L6
**chr14**	105055414	105055805	392	0,94	1,13E-02	5' UTR	GPR132
**chr4**	125364004	125364413	410	0,94	8,79E-02	Intron	FAT4
**chr1**	152329499	152329989	491	0,92	3,91E-02	Intron	FLG-AS1,FLG
**chr2**	26517832	26518165	334	0,91	5,87E-02	Intron	OTOF
**chr14**	78300899	78301296	398	0,91	5,93E-02	Intron	NRXN3
**chr14**	74760205	74760489	285	0,91	9,68E-02	Promoter	YLPM1
**chr3**	197065893	197066460	568	0,90	7,74E-02	Intron	DLG1
**chr4**	17405043	17405424	382	0,89	7,15E-02	Distal Intergenic	SNORA75B
**chr2**	138584288	138584965	678	0,89	7,65E-02	Distal Intergenic	SPOPL
**chr12**	131050488	131050816	329	0,88	2,34E-02	Intron	ADGRD1
**chr13**	48274225	48274634	410	0,87	5,93E-02	Distal Intergenic	RB1,ITM2B
**chr16**	80123355	80123905	551	0,86	5,07E-02	Intron	MAFTRR
**chr3**	154343227	154343625	399	0,85	5,61E-02	Intron	GPR149
**chr5**	125122782	125123235	454	0,84	7,71E-02	Intron	LINC02240
**chr17**	61402455	61403105	651	0,83	4,43E-02	3' UTR	TBX2-AS1,TBX2
**chr20**	59755679	59756225	547	0,82	7,74E-02	Intron	PHACTR3
**chr10**	11920866	11921394	529	0,81	9,23E-02	3' UTR	UPF2
**chr8**	70140129	70140489	361	0,78	5,93E-02	Intron	NCOA2
**chr4**	152596881	152597227	347	0,78	6,19E-02	Distal Intergenic	MIR4453
**chr21**	8464546	8465217	672	0,78	9,55E-02	Distal Intergenic	-
**chr1**	95060006	95060365	360	0,78	9,84E-02	Intron	ALG14,LOC101928098
**chr16**	25794953	25795544	592	0,75	8,68E-02	Intron	HS3ST4
**chr6**	123846716	123847097	382	0,69	6,79E-02	Intron	NKAIN2
**chr7**	45253163	45253643	481	0,67	7,65E-02	Distal Intergenic	RAMP3
**chr20**	34742322	34742774	453	0,66	7,71E-02	Exon	NCOA6
**chr15**	36909528	36910034	507	0,66	9,11E-02	Intron	MEIS2
**chr17**	39725183	39725612	430	0,61	9,97E-02	3' UTR	ERBB2,MIR4728,MIEN1
**chr15**	31791857	31792622	766	-0,54	6,79E-02	Intron	OTUD7A
**chr15**	29384204	29384946	743	-0,55	5,68E-02	Intron	LOC100130111
**chr12**	59328151	59328750	600	-0,62	4,50E-02	Distal Intergenic	SLC16A7
**chr2**	113330573	113330968	396	-0,63	4,87E-02	Distal Intergenic	PAX8
**chr15**	101438573	101439335	763	-0,63	9,97E-02	Intron	PCSK6
**chr2**	9909349	9909736	388	-0,64	9,65E-02	Intron	TAF1B
**chr3**	196098723	196100292	1570	-0,66	6,14E-02	Distal Intergenic	TFRC
**chr3**	13139948	13140350	403	-0,70	9,97E-02	Intron	IQSEC1
**chrX**	19543250	19543988	739	-0,74	2,34E-02	Intron	SH3KBP1

**chr6**	99710980	99711683	704	-0,75	5,21E-02	Distal Intergenic	PRDM13
**chr8**	21564253	21564668	416	-0,75	5,93E-02	Distal Intergenic	GFRA2
**chr6**	153860938	153861412	475	-0,77	1,51E-02	Distal Intergenic	OPRM1
**chr18**	11430292	11430865	574	-0,77	7,15E-02	Distal Intergenic	LINC01928
**chrX**	32357519	32358011	493	-0,77	7,71E-02	Intron	DMD
**chr4**	113983355	113983699	345	-0,78	9,68E-02	Distal Intergenic	ARSJ
**chr6**	38397668	38398082	415	-0,79	6,85E-02	Intron	BTBD9
**chr20**	40591720	40592100	381	-0,83	4,05E-02	Distal Intergenic	MAFB
**chr11**	8309097	8309445	349	-0,85	9,75E-02	Distal Intergenic	LMO1
**chr8**	29048909	29049484	576	-0,87	4,50E-02	3' UTR	HMBOX1
**chr3**	169667729	169668083	355	-0,87	6,67E-02	Distal Intergenic	MECOM
**chr12**	123891374	123891655	282	-0,88	6,37E-02	Intron	ZNF664
**chrX**	50185734	50186096	363	-0,89	4,55E-02	Distal Intergenic	AKAP4
**chrX**	132206020	132206380	361	-0,89	7,65E-02	Intron	RAP2C
**chrX**	7189691	7190082	392	-0,90	7,65E-02	Distal Intergenic	STS
**chrX**	101136018	101136420	403	-0,91	1,13E-02	Intron	CENPI
**chrX**	55069701	55070130	430	-0,91	7,15E-02	Distal Intergenic	PAGE2B
**chrX**	121681653	121682540	888	-0,92	4,80E-02	Distal Intergenic	MIR3672
**chrX**	18076768	18077182	415	-0,93	3,00E-02	Intron	LINC01456
**chr16**	69755715	69756075	361	-0,93	8,28E-02	Promoter	NOB1
**chrX**	150808754	150809078	325	-0,93	9,65E-02	Intron	CD99L2
**chrX**	22665040	22665413	374	-0,94	5,21E-02	Intron	PTCHD1-AS
**chr12**	68406085	68406642	558	-0,95	5,07E-02	Intron	LINC02384
**chr7**	72006797	72007150	354	-0,95	6,67E-02	Intron	CALN1
**chrX**	153415777	153416124	348	-0,95	6,96E-02	Promoter	ZFP92
**chrX**	93991247	93991572	326	-0,95	7,71E-02	Distal Intergenic	FAM133A
**chrX**	15685167	15685568	402	-0,95	7,88E-02	Intron	CA5BP1,CA5B
**chrX**	63959775	63960115	341	-0,95	9,33E-02	Distal Intergenic	ARHGEF9
**chr22**	35351420	35351749	330	-0,96	4,50E-02	Distal Intergenic	MIR6069,TOM1
**chr8**	77712716	77713039	324	-0,96	9,97E-02	Distal Intergenic	LOC102724874
**chrX**	9672381	9672868	488	-0,97	1,04E-02	Intron	TBL1X
**chr15**	34423023	34423365	343	-0,99	7,40E-03	Intron	MIR1233-2,GOLGA8A
**chr18**	76618556	76618929	374	-1,00	4,05E-02	Intron	LINC00683,LINC01927
**chrX**	138336585	138336959	375	-1,02	7,74E-02	Distal Intergenic	MIR504
**chr3**	39480691	39481132	442	-1,02	8,49E-02	Intron	MOBP
**chr8**	104348090	104348464	375	-1,03	7,40E-03	Intron	DPYS,DCSTAMP
**chr13**	109314084	109314469	386	-1,03	5,68E-02	Distal Intergenic	LINC00399
**chr14**	23925296	23925783	488	-1,03	5,69E-02	Intron	DHRS4
**chrX**	89762472	89762789	318	-1,04	6,37E-02	Distal Intergenic	TGIF2LX
**chr19**	30742950	30743417	468	-1,05	4,80E-02	Distal Intergenic	LINC01791
**chr8**	58401934	58402286	353	-1,05	5,07E-02	Distal Intergenic	UBXN2B
**chr11**	115562907	115563271	365	-1,07	1,41E-02	Distal Intergenic	CADM1
**chrX**	125801234	125801545	312	-1,08	4,50E-02	Distal Intergenic	LOC101928495
**chrX**	54245326	54245732	407	-1,08	6,85E-02	Intron	WNK3
**chrX**	13050487	13050922	436	-1,11	7,38E-03	Distal Intergenic	FAM9C

**chrX**	17812564	17812970	407	-1,12	2,50E-02	Intron	RAI2
**chrX**	67106461	67106783	323	-1,12	3,00E-02	Distal Intergenic	AR
**chr11**	105460681	105461081	401	-1,15	7,38E-03	Intron	CARD18
**chrX**	15529210	15529517	308	-1,17	1,51E-02	Intron	BMX
**chr6**	113752552	113752955	404	-1,18	6,67E-02	Distal Intergenic	LINC02541
**chrX**	74292249	74292667	419	-1,18	8,68E-02	Promoter	FTX,MIR374A
**chrX**	64248528	64248888	361	-1,19	7,74E-02	Distal Intergenic	ASB12
**chr7**	68650297	68650619	323	-1,20	5,07E-02	Distal Intergenic	LOC102723427
**chr4**	135169415	135170242	828	-1,22	7,38E-03	Distal Intergenic	LINC02485
**chr3**	26242309	26242695	387	-1,22	7,38E-03	Distal Intergenic	LINC00692
**chr10**	490113	490516	404	-1,22	8,79E-02	Intron	DIP2C
**chrX**	85929139	85929538	400	-1,26	1,82E-03	Intron	CHM
**chr10**	44662615	44663049	435	-1,27	6,93E-02	Distal Intergenic	TMEM72
**chrX**	124465548	124465914	367	-1,28	7,38E-03	Intron	TENM1
**chr8**	12484419	12484956	538	-1,29	7,38E-03	Intron	LOC729732
**chr9**	42740753	42741167	415	-1,39	7,65E-02	Distal Intergenic	LOC101927827
**chrX**	113447866	113448169	304	-1,41	6,79E-02	Distal Intergenic	AMOT
**chrX**	29379691	29380061	371	-1,42	1,46E-02	Intron	IL1RAPL1
**chr15**	21282529	21282914	386	-1,45	8,68E-02	Distal Intergenic	MIR3118-3,POTEB,MIR5701-3
**chr4**	60572610	60572978	369	-1,46	7,71E-02	Distal Intergenic	MIR548AG1
**chr20**	47832388	47832833	446	-1,53	7,71E-02	Distal Intergenic	SULF2
**chr1**	161458425	161459908	1484	-1,78	7,38E-03	Intron	FCGR2A
**chr10**	45732890	45733196	307	-1,79	2,35E-02	Intron	WASHC2C
**chr17**	46396140	46397322	1183	-1,93	7,15E-02	Intron	MIR4315-2
**chrX**	149731633	149732076	444	-1,95	5,19E-02	Distal Intergenic	MAGEA11
**chr3**	159681719	159682162	444	-1,96	3,01E-02	Intron	IQCJ-SCHIP1,SCHIP1
**chr22**	11837417	11837952	536	-2,03	9,68E-02	Distal Intergenic	FRG1FP
**chr13**	20119461	20119837	377	-2,07	4,50E-02	Distal Intergenic	LINC01072
**chr1**	25406068	25406495	428	-2,25	7,38E-03	Intron	RHCE
**chr9**	42600548	42600962	415	-2,46	7,38E-03	Distal Intergenic	LOC101927827
**chr17**	83232087	83232489	403	-3,02	7,40E-03	Intron	RPL23AP87
**chr11**	130438	131057	620	-3,40	7,70E-03	Exon	LINC01001
**chr14**	106757811	106758265	455	-4,35	7,38E-03	Distal Intergenic	MIR5195
**chr1**	228557690	228558459	770	-4,76	7,70E-03	Exon	BTNL10

DiffBind narrow window

Chr	start	end	width	log2fold	adj. p-value	annotation	associated genes
**chr16**	953843	953893	51	5,69	2,77E-05	Intron	LMF1
**chr1**	25292695	25292745	51	3,42	2,77E-05	Intron	RSRP1,RHD
**chr1**	25323341	25323391	51	3,19	1,13E-02	Intron	RSRP1,RHD
**chr9**	61944687	61944737	51	2,86	1,79E-03	Intron	FAM27C
**chr21**	13368336	13368386	51	2,86	1,21E-02	Distal Intergenic	-

**chr15**	30182939	30182989	51	2,53	4,51E-05	Distal Intergenic	LINC02249
**chr15**	48530201	48530251	51	2,47	1,55E-03	Intron	FBN1
**chr3**	128694830	128694880	51	2,40	1,13E-02	Distal Intergenic	RPN1
**chr9**	61867665	61867715	51	2,33	6,03E-02	Distal Intergenic	FAM27C
**chr2**	240680899	240680949	51	2,32	4,10E-02	Promoter	AQP12B
**chr11**	70345473	70345523	51	2,25	8,62E-02	Intron	PPFIA1
**chr20**	63234294	63234344	51	2,00	3,63E-02	Promoter	BIRC7,MIR3196
**chr16**	88501484	88501534	51	1,93	8,21E-02	Intron	ZFPM1
**chr4**	37585212	37585262	51	1,90	8,71E-02	Intron	C4orf19
**chr4**	120254207	120254257	51	1,79	5,20E-02	Intron	MAD2L1
**chr21**	8449677	8449727	51	1,71	5,20E-02	Distal Intergenic	FP236383.3-202
**chr10**	46860760	46860810	51	1,58	9,57E-02	Distal Intergenic	FRMPD2B
**chr7**	3299406	3299456	51	1,57	2,00E-02	Promoter	SDK1
**chr4**	119835902	119835952	51	1,56	5,07E-02	Distal Intergenic	LINC01365
**chr3**	103896484	103896534	51	1,55	7,49E-02	Distal Intergenic	MIR548AB
**chr10**	30328831	30328881	51	1,54	1,55E-03	Intron	MTPAP
**chr8**	109763044	109763094	51	1,52	3,63E-03	Distal Intergenic	SYBU
**chr16**	23748999	23749049	51	1,51	8,74E-02	Distal Intergenic	CHP2
**chr12**	64483999	64484049	51	1,49	1,71E-02	Exon	TBK1
**chr5**	26640171	26640221	51	1,48	8,06E-02	Distal Intergenic	CDH9
**chr14**	53150027	53150077	51	1,45	3,40E-02	5' UTR	DDHD1,LOC101927620
**chr2**	128921387	128921437	51	1,45	7,88E-02	Distal Intergenic	LINC01854
**chr1**	69099263	69099313	51	1,44	3,51E-02	Intron	LINC01707
**chr5**	89033162	89033212	51	1,43	7,67E-02	Intron	MEF2C-AS1
**chr10**	44613339	44613389	51	1,42	5,75E-02	Distal Intergenic	CXCL12
**chr12**	89438411	89438461	51	1,41	8,51E-02	Intron	POC1B
**chr16**	18286711	18286761	51	1,40	5,07E-02	Distal Intergenic	MIR6770-2
**chr4**	127548324	127548374	51	1,39	2,36E-02	Distal Intergenic	INTU
**chr21**	9666813	9666863	51	1,37	6,03E-02	Distal Intergenic	AC124864.2-201
**chr4**	80166018	80166068	51	1,37	8,21E-02	Distal Intergenic	PRDM8
**chr5**	157200085	157200135	51	1,35	2,23E-02	Intron	ITK
**chr14**	54439464	54439514	51	1,34	5,09E-02	Promoter	CNIH1
**chr2**	229208785	229208835	51	1,33	8,51E-02	Intron	PID1
**chr8**	37143004	37143054	51	1,30	8,51E-02	Distal Intergenic	KCNU1
**chr1**	58773156	58773206	51	1,29	5,22E-02	Distal Intergenic	JUN
**chr7**	109161739	109161789	51	1,26	9,57E-02	Distal Intergenic	C7orf66
**chr11**	109294678	109294728	51	1,25	8,71E-02	Distal Intergenic	C11orf87
**chr1**	80330845	80330895	51	1,25	9,32E-02	Distal Intergenic	LINC01781
**chr15**	94013994	94014044	51	1,25	9,85E-02	Intron	LINC01581,LINC01579
**chr9**	62039317	62039367	51	1,24	1,36E-02	Intron	FAM27C
**chr6**	28237372	28237422	51	1,23	3,40E-02	Distal Intergenic	ZSCAN9
**chr20**	56560762	56560812	51	1,23	7,13E-02	Distal Intergenic	LINC01716
**chr8**	117867342	117867392	51	1,23	7,65E-02	Intron	EXT1
**chr11**	113213998	113214048	51	1,20	4,69E-02	Intron	NCAM1
**chr5**	15454727	15454777	51	1,20	6,03E-02	Distal Intergenic	FBXL7

**chr3**	101839985	101840035	51	1,20	8,51E-02	Intron	NFKBIZ
**chr2**	39005876	39005926	51	1,19	5,20E-02	Intron	SOS1
**chr9**	1356734	1356784	51	1,19	5,20E-02	Distal Intergenic	DMRT2
**chr5**	165869569	165869619	51	1,17	4,24E-02	Distal Intergenic	LINC01947
**chr13**	36870392	36870442	51	1,17	8,33E-02	Intron	SMAD9
**chr20**	23316654	23316704	51	1,16	7,88E-02	Distal Intergenic	NXT1
**chr16**	66852529	66852579	51	1,16	8,51E-02	Intron	CA7,NAE1
**chr12**	26209786	26209836	51	1,15	6,51E-02	Intron	SSPN
**chr12**	12439761	12439811	51	1,15	8,91E-02	Intron	BORCS5
**chr17**	4540857	4540907	51	1,14	4,69E-02	Intron	MYBBP1A,SPNS2
**chr6**	14376962	14377012	51	1,12	4,77E-02	Distal Intergenic	LINC01108
**chr14**	99637138	99637188	51	1,08	1,13E-02	Distal Intergenic	HHIPL1
**chr5**	10132486	10132536	51	1,08	4,77E-02	Distal Intergenic	CCT5
**chr10**	61487853	61487903	51	1,07	2,36E-02	Distal Intergenic	TMEM26
**chr1**	152329703	152329753	51	1,07	5,20E-02	Intron	FLG-AS1,FLG
**chr13**	73209578	73209628	51	1,06	6,83E-02	Distal Intergenic	KLF5
**chr1**	12369197	12369247	51	1,06	7,29E-02	Intron	VPS13D
**chr9**	118140848	118140898	51	1,05	6,71E-02	Distal Intergenic	TLR4
**chr11**	87967848	87967898	51	1,05	9,32E-02	Intron	RAB38
**chr10**	109968603	109968653	51	1,04	4,96E-02	Intron	ADD3-AS1
**chr7**	42051973	42052023	51	1,04	5,20E-02	Intron	GLI3
**chr18**	3726434	3726484	51	1,03	1,61E-02	Intron	DLGAP1
**chr2**	176676925	176676975	51	1,01	4,27E-02	Distal Intergenic	LINC01116
**chr11**	94974765	94974815	51	1,01	9,91E-02	Promoter	KDM4D,CWC15
**chr1**	2325192	2325242	51	1,00	6,46E-02	Intron	MORN1
**chr19**	36121176	36121226	51	1,00	7,29E-02	Intron	TBCB
**chr11**	44061422	44061472	51	1,00	8,91E-02	Distal Intergenic	ACCS,ACCSL
**chr1**	243721055	243721105	51	0,99	8,51E-02	Intron	LOC339529
**chr10**	11920938	11920988	51	0,99	8,51E-02	3' UTR	UPF2
**chr3**	162717680	162717730	51	0,98	2,36E-02	Distal Intergenic	LINC01192
**chr11**	78451538	78451588	51	0,98	7,29E-02	Intron	NARS2
**chr12**	131050659	131050709	51	0,97	4,76E-02	Intron	ADGRD1
**chr2**	225212517	225212567	51	0,97	7,88E-02	Distal Intergenic	DOCK10
**chr2**	82820655	82820705	51	0,95	5,20E-02	Distal Intergenic	DHFRP3
**chr14**	105055545	105055595	51	0,94	4,65E-02	5' UTR	GPR132
**chr1**	95060117	95060167	51	0,94	4,99E-02	Intron	ALG14,LOC101928098
**chr2**	26517899	26517949	51	0,94	7,90E-02	Intron	OTOF
**chr18**	43085006	43085056	51	0,94	8,51E-02	Intron	RIT2
**chr3**	147397863	147397913	51	0,92	8,51E-02	Intron	LOC440982,ZIC1,ZIC4
**chr10**	91426572	91426622	51	0,91	9,54E-02	Intron	HECTD2
**chr12**	63539369	63539419	51	0,91	9,85E-02	Distal Intergenic	DPY19L2
**chr1**	234964627	234964677	51	0,90	6,81E-02	Intron	LOC101927851
**chr10**	65226781	65226831	51	0,89	7,65E-02	Distal Intergenic	LINC02671
**chr3**	154343440	154343490	51	0,88	9,85E-02	Intron	GPR149
**chr2**	95201133	95201183	51	0,81	4,77E-02	Distal Intergenic	ZNF2

**chr6**	34164970	34165020	51	0,81	7,36E-02	Distal Intergenic	GRM4
**chr19**	15532240	15532290	51	0,57	8,51E-02	Intron	CYP4F22
**chr2**	113330678	113330728	51	-0,69	8,33E-02	Distal Intergenic	PAX8
**chr20**	40591922	40591972	51	-0,83	7,88E-02	Distal Intergenic	MAFB
**chrX**	132206187	132206237	51	-0,86	9,78E-02	Intron	RAP2C
**chr15**	75783091	75783141	51	-0,89	3,50E-02	Exon	MIR4313
**chrX**	54415623	54415673	51	-0,89	5,20E-02	Distal Intergenic	TSR2
**chr12**	448691	448741	51	-0,89	9,32E-02	Distal Intergenic	B4GALNT3
**chr12**	123891529	123891579	51	-0,94	8,21E-02	Intron	ZNF664
**chr8**	104348301	104348351	51	-0,95	6,51E-02	Intron	DPYS,DCSTAMP
**chrX**	138975718	138975768	51	-0,95	7,88E-02	Intron	FGF13
**chrX**	6922993	6923043	51	-0,96	4,65E-02	Distal Intergenic	MIR4767
**chr12**	69032768	69032818	51	-0,96	9,21E-02	Distal Intergenic	CPM
**chr1**	11524600	11524650	51	-0,98	3,50E-02	Intron	DISP3
**chr3**	169667911	169667961	51	-0,99	7,67E-02	Distal Intergenic	MECOM
**chrX**	18076951	18077001	51	-1,00	6,46E-02	Intron	LINC01456
**chr15**	34423114	34423164	51	-1,00	7,67E-02	Intron	MIR1233-2,GOLGA8A
**chrX**	22665163	22665213	51	-1,02	8,91E-02	Intron	PTCHD1-AS
**chrX**	118513003	118513053	51	-1,02	9,32E-02	Intron	DOCK11
**chrX**	101136231	101136281	51	-1,03	3,69E-02	Intron	CENPI
**chr22**	35351526	35351576	51	-1,04	4,25E-02	Distal Intergenic	MIR6069,TOM1
**chrX**	151081257	151081307	51	-1,04	7,14E-02	Distal Intergenic	MIR4330
**chrX**	125801410	125801460	51	-1,05	9,23E-02	Distal Intergenic	LOC101928495
**chr12**	3368257	3368307	51	-1,06	2,24E-02	Promoter	LINC02417,LOC100128253
**chr8**	77712792	77712842	51	-1,07	9,54E-02	Distal Intergenic	LOC102724874
**chrX**	11192104	11192154	51	-1,08	6,03E-02	Intron	ARHGAP6
**chr16**	83659153	83659203	51	-1,09	4,76E-02	Intron	CDH13
**chr6**	14299541	14299591	51	-1,09	9,57E-02	Distal Intergenic	LINC01108
**chrX**	67106557	67106607	51	-1,10	3,69E-02	Distal Intergenic	AR
**chrX**	150808930	150808980	51	-1,10	6,03E-02	Intron	CD99L2
**chr3**	26623928	26623978	51	-1,11	7,14E-02	Promoter	LRRC3B
**chr18**	76618747	76618797	51	-1,12	2,33E-02	Intron	LINC00683,LINC01927
**chr11**	115563097	115563147	51	-1,12	7,70E-02	Distal Intergenic	CADM1
**chrX**	6945991	6946041	51	-1,14	6,03E-02	Distal Intergenic	MIR4767
**chr6**	99095971	99096021	51	-1,14	7,36E-02	Distal Intergenic	MIR548AI
**chrX**	55069834	55069884	51	-1,15	7,14E-02	Distal Intergenic	PAGE2B
**chr1**	37311910	37311960	51	-1,17	7,88E-02	Intron	MIR4255
**chr2**	140429152	140429202	51	-1,18	8,21E-02	Intron	LRP1B
**chr5**	153673200	153673250	51	-1,19	2,90E-02	Intron	GRIA1
**chrX**	152158838	152158888	51	-1,20	1,60E-02	Intron	MAGEA10
**chr5**	38060772	38060822	51	-1,22	9,36E-02	Intron	LINC02107
**chrX**	71541523	71541573	51	-1,24	1,79E-02	Intron	OGT
**chr1**	109256111	109256161	51	-1,24	2,36E-02	Intron	CELSR2
**chr4**	184113844	184113894	51	-1,25	3,40E-02	Intron	ENPP6
**chr12**	54945528	54945578	51	-1,25	3,40E-02	Distal Intergenic	TESPA1

**chrX**	85929243	85929293	51	-1,28	1,13E-02	Intron	CHM
**chrX**	89762645	89762695	51	-1,28	2,24E-02	Distal Intergenic	TGIF2LX
**chrX**	53882483	53882533	51	-1,28	7,88E-02	Distal Intergenic	PHF8
**chrX**	124465657	124465707	51	-1,29	3,18E-02	Intron	TENM1
**chrX**	151248678	151248728	51	-1,32	3,40E-02	Distal Intergenic	GPR50-AS1
**chrX**	16229890	16229940	51	-1,33	6,66E-02	Distal Intergenic	MAGEB17
**chr17**	52837101	52837151	51	-1,34	2,29E-02	Distal Intergenic	MIR4315-2
**chr8**	59459615	59459665	51	-1,34	6,03E-02	Distal Intergenic	TOX
**chrX**	6733191	6733241	51	-1,34	8,09E-02	Distal Intergenic	VCX3A
**chr7**	153925447	153925497	51	-1,36	7,14E-02	Intron	DPP6
**chr7**	71093710	71093760	51	-1,37	1,13E-02	Distal Intergenic	GALNT17
**chr7**	68650369	68650419	51	-1,40	4,10E-02	Distal Intergenic	LOC102723427
**chr8**	58402076	58402126	51	-1,40	5,20E-02	Distal Intergenic	UBXN2B
**chrX**	15220693	15220743	51	-1,40	6,51E-02	Distal Intergenic	ASB9
**chr21**	42258091	42258141	51	-1,40	8,91E-02	Intron	ABCG1
**chr2**	1706584	1706634	51	-1,40	9,88E-02	Intron	PXDN
**chr11**	105460895	105460945	51	-1,41	8,40E-03	Intron	CARD18
**chr13**	60803287	60803337	51	-1,42	8,41E-02	Distal Intergenic	LINC00378
**chr3**	26242510	26242560	51	-1,43	8,40E-03	Distal Intergenic	LINC00692
**chr8**	12484674	12484724	51	-1,43	3,03E-02	Intron	LOC729732
**chr18**	33067535	33067585	51	-1,45	7,14E-02	Intron	CCDC178
**chrX**	29379913	29379963	51	-1,47	9,32E-02	Intron	IL1RAPL1
**chr4**	60572869	60572919	51	-1,51	7,65E-02	Distal Intergenic	MIR548AG1
**chr4**	135170099	135170149	51	-1,52	8,51E-02	Distal Intergenic	LINC02485
**chr22**	42072946	42072996	51	-1,53	2,48E-02	Promoter	PHETA2,NAGA
**chrX**	42915747	42915797	51	-1,54	8,74E-02	Distal Intergenic	PPP1R2C
**chr8**	101985976	101986026	51	-1,56	1,79E-02	Intron	NCALD
**chr15**	74702200	74702250	51	-1,56	9,67E-02	Distal Intergenic	EDC3
**chr22**	40185897	40185947	51	-1,62	1,60E-02	Intron	TNRC6B
**chr7**	154662123	154662173	51	-1,71	1,61E-02	Intron	DPP6
**chr8**	128316307	128316357	51	-1,71	4,69E-02	Distal Intergenic	MIR1208
**chr20**	47832617	47832667	51	-1,73	3,15E-02	Distal Intergenic	SULF2
**chr12**	4758443	4758493	51	-1,76	8,39E-03	Intron	GALNT8,NDUFA9
**chr1**	161459262	161459312	51	-1,84	1,13E-02	Intron	FCGR2A
**chr8**	3323938	3323988	51	-1,85	2,36E-02	Intron	CSMD1
**chr4**	146113657	146113707	51	-1,94	3,40E-02	Exon	LINC01095
**chr17**	40892676	40892726	51	-1,95	1,36E-02	Distal Intergenic	KRT10
**chr16**	54095151	54095201	51	-2,00	7,88E-02	Intron	FTO
**chr10**	45732942	45732992	51	-2,05	1,60E-02	Intron	WASHC2C
**chr15**	52254525	52254575	51	-2,11	3,18E-02	Intron	MYO5C
**chr13**	20119566	20119616	51	-2,19	5,46E-03	Distal Intergenic	LINC01072
**chr2**	236254404	236254454	51	-2,24	8,09E-02	Intron	ASB18
**chr1**	25406267	25406317	51	-2,31	3,22E-04	Intron	RHCE
**chr4**	189836541	189836591	51	-2,62	8,51E-02	Intron	DBET
**chr17**	83232374	83232424	51	-2,71	6,03E-02	Intron	RPL23AP87

**chr14**	106757931	106757981	51	-3,93	1,13E-02	Distal Intergenic	MIR5195

Supplementary Table S2. Primers used in the RT-PCR analysis.

Gene	Primers	Annealing temperature (°C)
TET1	F: 5′AAATGTTGCCCGAGAATGTC3′<br>R: 5′TGTTGTGAATGTCCCTGTGG3′	56
TET2	F: 5′GAAGGAATCCCGCTGTCTC3′<br>R: 5′ACAGGCGCAAGTTCTCTCTT3′	56
TET3	F: 5′CTGGAGCATGTACTTCAACG3′<br>R: 5′5′ACGGCAGTCAATCGCTATTTC3′	56
CHM	F: 5′GAAATGATAATGCAGTCAAACAGG3′<br>R: 5′GTGGAGGGGGACAGAAATC3′	60
MTPAP	F: 5′ACGATTTCAGCTATAGCCCACT3′<br>R:5′GCTAATTCTCAAATTTTCTAAAGATGG3′	60
TMEM50A	F: 5′AGACCAGCACGGTCAACTAGA3′<br>R: 5′TTCTGAAGAGGAAGAGACAACAAG3′	61
RSRP1	F: 5′TTGACTTGCCAGCTAGTCTCAG3′<br>R: 5′TTCTGTTACCTTTTCCGACAGTT3′	60
TBP	F: 5′ACTCCACTGTATCCCTCCC3′<br>R: 5′TATATTCGGCGTTTCGGGCA3′	60

F: forward. R: reverse.
